# *Vulcanimicrobium alpinus* gen. nov. sp. nov., the first cultivated representative of the candidate phylum “Eremiobacterota”, is a metabolically versatile aerobic anoxygenic phototroph

**DOI:** 10.1038/s43705-022-00201-9

**Published:** 2022-12-16

**Authors:** Shuhei Yabe, Kiyoaki Muto, Keietsu Abe, Akira Yokota, Hubert Staudigel, Bradley M. Tebo

**Affiliations:** 1 Department of Microbial Resources, Graduate School of Agricultural Sciences, Tohoku University, Sendai, Miyagi 980-0845 Japan; 2 Hazaka Plant Research Center, Kennan Eisei Kogyo Co., Ltd., Sendai, Miyagi 989-1311 Japan; 3 Institute of Geophysics and Planetary Physics, Scripps Institution of Oceanography, University of California San Diego, La Jolla, CA 92093 USA; 4 Department of Chemistry, University of Washington, Box 351700, Seattle, WA 98195 USA

**Keywords:** Soil microbiology, Microbial ecology

## Abstract

The previously uncultured phylum “*Candidatus* Eremiobacterota” is globally distributed and often abundant in oligotrophic environments. Although it includes lineages with the genetic potential for photosynthesis, one of the most important metabolic pathways on Earth, the absence of pure cultures has limited further insights into its ecological and physiological traits. We report the first successful isolation of a “*Ca*. Eremiobacterota” strain from a fumarolic ice cave on Mt. Erebus volcano (Antarctica). Polyphasic analysis revealed that this organism is an aerobic anoxygenic photoheterotrophic bacterium with a unique lifestyle, including bacteriochlorophyll *a* production, CO_2_ fixation, a high CO_2_ requirement, and phototactic motility using type IV-pili, all of which are highly adapted to polar and fumarolic environments. The cells are rods or filaments with a vesicular type intracytoplasmic membrane system. The genome encodes novel anoxygenic Type II photochemical reaction centers and bacteriochlorophyll synthesis proteins, forming a deeply branched monophyletic clade distinct from known phototrophs. The first cultured strain of the eighth phototrophic bacterial phylum which we name *Vulcanimicrobium alpinus* gen. nov., sp. nov. advances our understanding of ecology and evolution of photosynthesis.

## Introduction

Geobiology, the discipline that concerns itself with interactions between the Earth’s biosphere and lithosphere and its evolution through geologic time, has made great advances in our knowledge of oligotrophic lithospheric systems, with molecular studies showing the genetic diversity and metabolic potential of microbial consortia as well as unraveling important microbial biogeochemical and metabolic functions in laboratory systems [1]. However, it has been notoriously difficult to isolate the microbes from natural communities that are necessary to explore diverse microbial processes, how these processes actually function, how they may depend on environmental conditions and how they may have evolved in geological history [2, 3]. While these are quite far-reaching scientific goals, isolating difficult to cultivate organisms, especially those found widespread in oligotrophic environments, is important to advance our understanding of geobiology. For example, oligotrophic lithospheric environments likely played a key role in where and how early life on earth evolved and isolation of key organisms would enable their interrogation under realistic conditions in the laboratory.

An uncultured phylum “*Ca*. Eremiobacterota” (formerly known as WPS-2 or WD272) is an ecologically versatile phylum that apparently thrives under various oligotrophic environments [4], and includes lineages with the potential for photosynthesis [5, 6], and a novel form of chemolithoautotrophy called “atmospheric chemosynthesis” [4, 7, 8]. To date, only “*Ca*. Eremiobacterota” among seven currently recognized phototrophic phyla (*Cyanobacteria*, *Chlorobiota*, *Actinomycetota*, *Firmicutes*, *Proteobacteria*, *Chloroflexota*, and *Gemmatimonadota* [9–13]) remains uncultured. The ecological importance of this lineage has rendered it a targeted uncultured phylum given prioritization for cultivation attempts among the recent explosion of candidate phyla for characterization [14].

“*Ca*. Eremiobacterota” (WPS-2) was first described in 16 S rRNA gene clone libraries from polychlorinated biphenyl-contaminated soil (Wittenberg-Polluted Soil) in Wittenberg, Germany [15], and the phylum name was proposed based on the metagenome-assembled genome (MAG) recovered from Antarctic soil [7]. Recent metagenomic studies revealed that “*Ca*. Eremiobacterota” is globally distributed in terrestrial [4] and marine environments [16], animal sources [17] and industrial wastes [17]; however, it is abundant in Antarctic bare soils [7, 18], Arctic permafrost soils [19], boreal mosses [6], and volcanic soils [20].

Presently (Sep 27 2022), “*Ca*. Eremiobacterota” comprise three class-level lineages; “*Ca*. Eremiobacteria” [5], “*Ca*. Xenobia” [4], and “*Ca*. Eudoremicrobiia” [16]. There are two representative order-level taxonomic groups within “*Ca*. Eremiobacteria”, “*Ca*. Eremiobacterales” [5] and “*Ca*. Baltobacterales” [5], each of which contain a single family-level group, “*Ca*. Eremiobacteraceae” and “*Ca*. Baltobacteraceae”, respectively [5]. “*Ca*. Eremiobacterales” includes two candidate genera, and “*Ca*. Baltobacterales” includes 16 candidate genera [4]. Several lineages within “*Ca*. Baltobacterales” have a new family of anoxygenic Type II photochemical reaction centers (RCs) that are phylogenetically and structurally distinct from those found in known phototrophs [5, 6].

The fumarolic ice caves near the summit of Mt. Erebus (elevation 3794 m), a volcano on Ross Island, Antarctica, comprise one of the most remote dark oligotrophic volcanic ecosystems on the Earth, with minimal human-derived contamination. The ice caves, comprising volcanic rock and weathered sediments, are nutrient-poor, with 0–0.013% (w/w) of total organic carbon [21], a range comparable to that of the high-altitude volcanoes above 6000 m in the Atacama desert, reported being the best Earth analog for the surface of Mars [22]. Volcanic gases circulating through the cave atmosphere from vents under the glacier provide a CO_2_-rich and warm environment conducive for the growth of microorganisms [21]. A previous study of two completely dark ice caves and one cave with seasonally snow-filtered light using the PCR clone library method [21] revealed that the ice caves contain a high proportion of uncultured members of the “*Ca*. Eremiobacterota” with a 33.3% relative abundance, represented by OTU ID #HD1(WD272), #HD2, #HD29, and #HD30 in reference [17].

We here report the successful isolation, lifestyle, and physiological, genomic, and cell structural characterization of a new phototrophic bacterium belonging to “*Ca*. Eremiobacterota” from Warren Cave, a dark fumarolic ice cave in Antarctica. Its potential ecological roles are also discussed.

## Materials and methods

### Sampling, bacterial isolation, and colony screening

Samples were collected from thermally active sediments as evidenced by temperature and/or emission of steam in November 2010 and 2012 from Harry’s Dream (HD2 and HD3), Warren Cave (WC1, WC2, WC3, WC4, WC7, and WC8), Haggis Hole, (HH), Mammoth Cave (MC), Hut Cave (Hut), and Heroine Cave (HC) (fumarolic ice caves on Mt. Erebus volcano [21]). The samples were divided into four types: Mainly weathered basaltic/phonolitic sand (HD2, HD3, WC3, WC4, WC8 and Hut); pebbles and rock fragments (WC7, HH, and MC); black porous glassy materials that appeared to be solidified lava (WC1, WC2, and HC); and ash sediment (WC10) with little to no organic material (Table S1). The sediments were collected aseptically using sterile 50 mm conical tubes and immediately sealed. Additional information and environmental parameters on the sampling locations are provided in Table S1 and in Tebo et al. [21].

To isolate bacteria found in these oligotrophic environments, we employed a culture strategy of long-term incubation in a nutrient-poor medium and screening of slow-growing colonies by direct PCR identification. Reasoner’s 2 A gellan gum medium (10% R2AG) [23] and FS1VG medium [24] were used for bacterial isolation. The 10% R2AG is a 10-fold diluted R2A broth (Nihon Seiyaku, Tokyo, Japan), solidified with 15 g/L gellan gum (Kanto chemical, Tokyo, Japan) with 2 g/L CaCl_2_. The 10% R2AG and FS1VG media were adjusted to a pH of 4.5 or 6.0, with or without 30 mg/L sodium azide. Samples used for isolation were selected from Warren Cave and Harry’s Dream, where the presence of relatively large numbers of bacteria (>10^7^/g) was confirmed in a previous report [21], and a total of nine samples, WD1, 2, 3, 4, 7, 8, and 10 and HD2 and 3, were used without any other pretreatment such as drying or dilution. For the sandy and ash samples with fine particles (WC3, 4, 8, 10, and HD2, 3), approximately 50 mg of sample were spread directly on plates. Glassy materials (WC1 and WC2) were embedded directly in plates using 2-3 pieces (approx. 200 mg). Pebbles and rock fragments (WC7) were crushed and approximately 50 mg of debris were spread directly onto the plates. All plates were incubated at 15 °C, 30 °C or 37 °C under dark conditions. New colonies were marked as they appeared and selection of the isolates was performed by picking only colonies that appeared after four weeks of incubation. We selected colonies around the sediment or in the cracks created during spreading using a magnifying glass.

For identification, colonies were picked with a sterile toothpick, re-streaked, stabbed on fresh medium, and subsequently suspended in 20 μL sterilized 0.05 M NaOH. Suspensions were heated at 100 °C for 15 min, and supernatants were used as template DNAs for PCR. Partial 16 S rRNA gene sequences were amplified by PCR using commonly used bacterial primer set 27 F (5′-AGATTTGATCCTGGCTCAG-3′) and 1492 R (5′-GGTTACCTTGTTACGACTT-3′) or 536 R (5′-GTA TTA CCG CGG CTG CTG-3′) with TaKaRa ExTaq DNA polymerase (Takara Bio, Shiga, Japan) as previously described [23]. Sequencing was performed at Eurofins Genomics (Louisville, KY, USA), using a 3730xl DNA analyzer (Applied Biosystems, CA, USA). Sequence similarities with closest species were calculated using EZbiocloud’s Identify Service (https://www.ezbiocloud.net/identify). Subsequently, cells in the stab identified as “*Ca*. Eremiobacterota” by the direct PCR identification were serially diluted and stabbed onto new plates until multiple pure cultures of Eremiobacterota were obtained. The isolate was designated as strain WC8-2.

### Whole genome sequencing and annotation

Genomic DNA was extracted from WC8-2 cells grown in 10% R2A broth (pH6.0) with air/CO_2_ (90:10, v/v) at 30 °C for 30 days under a 12/12 h light/dark regime with incandescent light (250 μmol m^–2^s^–1^), using a Puregene Yeast/Bact. Kit B (Qiagen, Germantown, MD, USA) [25]. Sequencing was performed by Macrogen Japan Corp., on a NovaSeq 6000 (Illumina, Inc., San Diego, CA, USA) and PacBio RSII (Pacific Biosciences of California, Inc. Menlo Park, CA, USA). The gap-free complete genome was assembled de novo using the Unicycler version 0.4.8 hybrid assembly pipeline with default settings [26]. Completeness and contamination levels were estimated using CheckM [27]. The genome was annotated using the DDBJ Fast Annotation and Submission Tool (DFAST) [28] and the BlastKOALA web server version 2.2, and was visualized using CGView Server [29] (http://cgview.ca/).

### Phylogenetic analysis of “*Ca*. Eremiobacterota”

Identification of strain WC8-2 was performed using the Genome Taxonomy Database Toolkit (GTDB-Tk) (ver. 2.1.0), which produces standardized taxonomic labels that are based on those used in the Genome Taxonomy Database [30]. Terrabacterial genomes including “*Ca*. Eremiobacterota” MAGs and related genomes were retrieved from the Genome Taxonomy Database (GTDB) (July 2022) and the NCBI RefSeq database (July 2022). Full-length 16 S rRNA gene sequences were retrieved from the WC8-2 genome (WPS_r00030) and the NCBI database (Table S2). Multiple sequences were aligned using SINA (version 1.2.11) [31]. IQ-TREE version 1.6.12 [32] was used to build the phylogeny. ModelFinder [33] was used to determine the optimal evolutionary model for phylogeny building, which selected the TNe+I + G4 model. Branch support was calculated using 1000 ultrafast bootstraps [34]. The pairwise 16 S rRNA gene sequence similarities were determined using SDT software. Phylogenomic analysis based on 400 marker proteins was carried out using PhyloPhlAn v3.0 [35]. Diamond v5.2.32 [36], MAFFT v7.453 [37], and TrimAI were utilized for orthologs searching, multiple sequence alignment within the superphylum Terrabacteria, and gap-trimming, respectively. Gappy sites and sequences with >50% gaps were deleted from the alignments. IQ-TREE version 1.6.12 [32] was used to build the phylogenomic tree. ModelFinder [33] was used to determine the optimal evolutionary model for phylogeny building, which selected the LG + F + R9 model. These analyses were conducted using the “AOBA-B” super- computer (NEC, Tokyo, Japan) with 2CPUs (EPYC7702, AMD, CA, US) and 256GB of RAM. The related similarity of genomes between strain WC8-2 and relatives was estimated using average nucleotide identity (ANI) values, which were calculated using OrthoANI calculator in the EzBio-Cloud web service [38]. The related similarity between strain WC8-2 and its sister phyla with one representative from each class was assessed by pairwise Average Amino acid Identity (AAI) values using the online tools at the Kostas Konstantinidis Lab website Environmental Microbial Genomics Laboratory (http://enve-omics.ce.gatech.edu/aai/). The MAGs used in the tree are listed in Table S3.

### Phylogeny of photosynthesis- and “atmospheric chemosynthesis”- associated genes

We retrieved phototrophy- and “atmospheric chemosynthesis”-related protein sequences from the WC8-2 genome, “*Ca*. Eremiobacterota” MAGs, and known phototrophs genomes (Table S3) using the local BLAST server (SequenceServer 1.0.14 [39]) with reference sequences (Table S3) or annotated sequences as queries. Sequences were aligned using MUSCLE and poorly aligned regions were removed using Gblocks version 0.91b [40] or by manual inspection. The alignment sequences were concatenated into a single sequence. The ML tree was constructed using IQ-TREE with the best-hit evolutionary rate model: LG + I + G4 for HhyL, CbbL, BchXYZ and CbbL, LG + F + I + G4 for BchLNB, and LG + F + G4 for PufML, BchI, and BchD. All trees were visualized using iTOL (version 5.0) [41]. All sequences used in the trees are listed in Table S3.

### Analysis of bacterial communities in the fumarolic ice caves

Environmental DNA was extracted from the samples (0.1 g) (WC7, WC8, HD3, MC, Hut, HH, HC) using a DNeasy PowerSoil Kit (Qiagen, Valencia, CA, USA) following the manufacturer’s instructions. The V4 region of the 16 S rRNA gene was PCR amplified using primers with adapter sequences (V3-V4f_MIX) [42]. The PCR cycling was carried out using the following parameters: 94 °C for 2 min followed by 25 cycles at 94 °C for 30 s, 56 °C for 30 s, and 72 °C for 1 min with a final extension at 72 °C for 2 min. Library construction and sequencing were performed at the Bioengineering Lab (Kanagawa, Japan) using MiSeq (Illumina). Briefly, adaptor and primer regions were trimmed using the FASTX-Toolkit v0.0.13 (http://hannonlab.cshl.edu/fastx_toolkit). Read sequences of ≤40 bp with ambiguous bases and low-quality sequences (quality score, ≤Q20), together with their paired-end reads, were filtered out using Sickle v1.33 (https://github.com/najoshi/sickle). High-quality paired-end reads were merged using PEAR v0.9.10 with default settings [43]. Merged sequences of ≤245 and ≥260 bp were discarded using SeqKit v0.8.0 [44]. Operational taxonomic units (OTUs) were classified using QIIME v1.9.1 and the SILVA database (release 132) with 97% identity. To study the phylogeny of the OTUs assigned to the “*Ca*. Eremiobacterota”, a neighbor-joining (NJ) tree of the OTUs was constructed [45] as described above.

### Microscopic observation

Electron microscopic observations of the cells were performed at Tokai Electron Microscopy (Nagoya, Japan). Briefly, the cells grown in 10% R2A broth (pH6.0) with air/CO_2_ (90:10, v/v) at 30 °C for 15 days under a 12/12 h light/dark regime with incandescent light (250 μmol m^–2^s^–1^) were fixed with 4% paraformaldehyde (PFA) and 4% glutaraldehyde (GA) in 0.1 M phosphate buffer (PB) at pH7.4, and postfixed with 2% OsO_4_ in 0.1 M PB. Cells were then dehydrated using graded ethanol solutions. The dehydrated cells were polymerized with resin, ultrathin sectioned, stained with 2% uranyl acetate, then secondary-stained with Lead stain solution. A transmission electron microscope (TEM) (JEM-1400Pus; JEOL, Tokyo, Japan) was used to observe the ultrathin sectioned cells at 100 kV acceleration voltage. To observe the negative-stained cells, PFA- and GA-fixed cells were adsorbed on formvar film-coated copper grids and stained with 2% phosphotungstic acid solution (pH 7.0) and observed using a TEM at 100 kV. DAPI (4,6-diamidino2-phenylindole) and Nile-Red staining was performed by incubating 0.1 mL cell suspension with a 1 mL staining solution (1 mg/L DAPI and 1 mg/L Nile Red in PBS buffer) for 10 min. The stained cells were observed under a fluorescence microscope (Olympus AX80T; Olympus Optical; Tokyo, Japan).

### Growth assay

Unless otherwise noted, all cultures were grown in 100 mL butyl stopper- and screw-cap-sealed glass vials containing 50 mL liquid medium (pH6.0) at 30 °C. Growth was monitored by optical density at 600 nm (OD_600_) using a spectrophotometer (BioSpectrometer Basic; Eppendorf; Tokyo, Japan). Initial cell density was adjusted to 0.005 (OD_600_). Specific culture conditions are described below. All anaerobic growth tests were conducted with 100% N_2_ gas in the headspaces and supplemented with a reducing agent (0.3 g/L cysteine-HCl) and a redox indicator (1 mg/L resazurin).

#### Optimal culture conditions

Cell growth in different media was examined using 1, 10, 20, 100% (a full strength) R2A broth and Basal_YE with air/CO_2_ (90:10, v/v) under a 12/12 h light/dark regime with incandescent light (250 μmol m^–2^s^–1^) for 25 days. Basal_YE contained (l^−1^) 0.44 g KH_2_PO_4_, 0.1 g (NH_4_)_2_SO_4_, 0.1 g MgSO_4_.7H_2_O, 0.3 g yeast extract, and 1 ml trace element SL-8 [refer to DSMZ745]. Cell growth at different temperatures (10, 13, 20, 25, 30, 33 and 37 °C; pH6.0), pH (3.7, 4.5, 6.0, 7.0, 8.0, and 9.0), and NaCl (0, 1, 10, 20, and 30 g [l^−1^]; pH6.0) was examined using Basal_YE with air/CO_2_ (90:10, v/v) for 25 days under a 12/12 h light/dark regime with incandescent light (250 μmol m^−2^s^−1^). The following pH buffer solutions were used: acetic acid/sodium acetate for pH 4–6, K_2_HPO_4_/KH_2_PO_4_ for pH 6–8, sodium bicarbonate/sodium carbonate for pH 9–10. To examine colony formation on solid media, WC8-2 was streaked or stabbed on 10% R2AG medium (pH 6.0) and incubated at 30 °C for 30 days under a 100% air atmosphere.

#### Optimal oxygen and carbon dioxide conditions

To determine the preferred O_2_ concentration, WC8-2 was grown in Basal_YE (pH 6.0, 30 °C, no NaCl) in butyl stopper-sealed glass bottles with the atmosphere in the headspace adjusted to different N_2_/O_2_/CO_2_ ratios (70:20:10%, 80:10:10%, 89:1:10%, 90:0:10% v/v) after removing oxygen with 100% N_2_ gas. Cultures were incubated for 25 days under a 12/12 h light/dark regime with incandescent light (250 μmol m^–2^s^–1^). To determine the CO_2_ preference, the gas phase was adjusted to different N_2_/O_2_/CO_2_ ratios (70:20:10%,75:20:5%, 80:20:0% v/v) in sealed bottles or 100% air (plugged with a BIO-SILICO N-38 sponge plug; Shin-Etsu Polymer Co., Ltd, Tokyo, Japan; breathable culture-plug) under the same conditions as the O_2_ preference test.

#### Photoorganoheterotrophic (or photoorganoautotrophic) and chemoorganoheterotrophic (or chemoorganoautotrophic) growth

The utilization of organic compounds as carbon sources/organic electron donors was tested in Basal medium (Basal_YE without yeast extract, pH 6.0) supplemented with one of the following sources (l^-1^): 0.3 ml of glycerol, or 0.3 g of sucrose, d-glucose, d-ribose, maltose, l-leucine, l-isoleucine, l-valine, l-serine, l-lysine, taurine, yeast extract or gellan gum, 1 ml of vitamin B12 solution (2 mg/L). Utilization was assessed by measuring growth of the cultures during a 25-day incubation at 30 °C in continuous light (250 μmol m^–2^s^–1^) for photoorganoheterotrophy, or continuous dark for chemoorganoheterotrophy under aerobic (air/CO_2_ [90:10, v/v]) and anaerobic (N_2_/CO_2_ [90:10, v/v]) conditions.

#### Photolithoautotrophic and chemolithoautotrophic growth

Cells were inoculated into amended PSB2 [46] as described below or Basal medium with 5 mM Na_2_S or Na_2_S_2_O_3_ or 1% H_2_ (v/v; in the gas phase) as electron donors, and cultivated in continuous light (250 μmol m^–2^s^–1^) for photolithoautotrophic growth, or continuous dark for chemolithoautotrophic growth under aerobic (air/CO_2_ [90:10, v/v]) and anaerobic (N_2/_CO_2_ [90:10, v/v]) conditions for 60 days at 30 °C. The amended PBS2 contained (L-1): 0.5 g NH_4_Cl, 1.0 g KH_2_PO_4_, 0.2 g NaCl, 0.4 g MgSO_4_._7_H_2_O, 0.05 g CaCl_2_.2H_2_O, 4.2 g NaHCO_3_, 1 ml trace element SL-8 [refer to DSMZ745], and 1 ml vitamin B12 solution (2 mg/L), pH6.0.

#### Fermentative or anaerobic growth

Anaerobic growth was examined in continuous dark in 20% R2A broth (pH 6.0) with N_2_/CO_2_ (90:10, v/v) supplemented with 5 mM Na_2_SO_4_, NaNO_3_, or dimethyl sulfoxide (DMSO) as electron acceptors for 60 days at 30 °C.

### Pigment assays

Cells grown in Basal_YE (pH6.0) with air/CO_2_ (90:10, v/v) at 30 °C for 14 days (exponential growth phase) under continuous light (250 μmol m^–2^ s^–1^) and continuous dark were used for the pigment assays. The absorption spectrum was determined in a cell suspension in 60% (w/v) sucrose and in a 100% methanol extract using a spectrophotometer (V-630; JASCO, Tokyo, Japan) at 350–1100 nm. The BChl *a* concentration was determined spectroscopically in 100% methanol [47]. Dry cell weight was measured after harvested cells were washed twice with Milli-Q water and dried at 80 °C for 3 days. The extract was also analyzed by HPLC (NEXERA X2; Shimadzu; Kyoto, Japan) equipped with a 4.6 × 250 mm COSMOSIL 5C18-AR (Nakarai Taque; Tokyo, Japan) with isocratic elution of 92.5% (v/v) methanol in water at a flow rate of 1.0 mL/min. BChl *a* was monitored at 766 nm using a diode-array spectrophotometer detector (SPD-M20A; Shimadzu; Kyoto, Japan).

### Observation of taxis

To study phototaxis in WC8-2, cells were grown in 20% R2A broth with air/CO_2_ (90:10, v/v) for 14 days under a 12/12 h light/dark regimen. Cultures were transferred to tissue culture flasks (175 cm^2^, canted neck, Iwaki, Shizuoka, Japan). Light sources were ultraviolet (UV) at 395 nm (Linkman, Fukui, Japan), blue at 470 nm (CREE, Durham, NC, USA), green at 502 nm (Linkman), red at 653 nm (LENOO, Shinpei, Taiwan), and near-infrared (NIR) at 880 nm (LENOO). The light-emitting device was constructed by assembling LEDs on a breadboard with a power supply. Cultivations were illuminated with each wavelength using a light-emitting device from the underside and incubated at 20 °C for 18 h. Cells aggregating toward a light source was taken to indicate phototaxis. As a control, culture vessels were wrapped in aluminum foil to block light. Images and time-lapse video were captured using an iPhone 6 S camera.

### Stable carbon isotope ratio mass spectrometry (IRMS)

The WC8-2 cells were grown in Basal_YE (pH6.0) with air/^13^CO_2_ [90:10, v/v] and air/unlabeled CO_2_ [90:10, v/v] under continuous light (250 μmol m^–2^s^–1^) for photoheterotroph and continuous dark for chemoheterotroph at 30 °C for 14 days (exponential growth phase). Approximately 10 mg culture biomass was collected, washed in HCl overnight, rinsed three times with deionized water, and placed into tin capsules. Stable carbon isotope ratios (δ^13^C) were analyzed at Shoko Science (Saitama, Japan) using a Delta V Advantage (EA-IRMS; Thermo Fisher Scientific, Bremen, Germany). The standard for C isotope ratio analysis was Vienna PeeDee Belemnite (VPDB). The δ^13^C values of ^13^CO_2_-cultivated cells exceeded the optimum calibration range of the instrument, but were used in this study to provide conclusive evidence that inorganic carbon was incorporated into the biomass.

### Quantitative reverse transcription PCR (qRT-PCR)

#### Total RNA extraction and cDNA synthesis

Total RNA was extracted from cells grown in Basal_YE (pH6.0) with air/CO_2_ [90:10, v/v] in continuous light (250 μmol m^–2^s^–1^) for photoheterotrophic conditions and in continuous dark for chemoheterotrophic conditions at 30 °C for 14 days (exponential growth phase) using the Total RNA Purification Kit (Norgen, Biotek Corp, Ontario, Canada). DNA was removed from the extracted nucleic acids using an RNase-Free DNase I Kit (Norgen, Biotek Corp) according to the manufacturer’s protocol. The absence of DNA in the RNA samples was confirmed by PCR without reverse transcriptase. cDNA was generated from 500 ng total RNA using a TaKaRa PrimeScript™ 1st strand cDNA Synthesis Kit (TaKaRa Bio) with random hexamers according to the manufacturer’s protocol.

#### Primer design, specificity and efficiency

The following three photosynthesis- and CO_2_-fixation-related genes in the WC8-2 genome were selected for qRT-PCR: *bchM* encoding an enzyme involved in BChl synthesis, *pufL* encoding the anoxygenic Type II photochemical reaction centers L-subunit, and *cbbL* encoding the large subunit of type IE RuBisCO. The RNA polymerase subunit beta (*rpoB*) was used as a housekeeping reference gene. Primers for qRT-PCR were designed with Primer3 (v. 0.4.0) [48] with the following criteria: product size ranging from 80 to 150 bp, optimum Tm of 60 °C and GC content about 50 to 55%. Standard RT-PCR confirmed that each primer set amplified only a single product with expected size (data not shown), and the product was also sequenced using Sanger sequencing at Macrogen Japan Corp to confirm the candidate products. Primer efficiency was calculated for qRT-PCR using the slope of the calibration curve based on a 20-, 40-, 80-, 160-fold dilution series of cDNA samples [49]. In addition, the specificity of the primers was determined by the confirmation of a single peak in the melting curve. All information about the primers is shown in Table S4.

#### qRT-PCR

qRT-PCR was performed using a MiniOpticon Real-Time PCR System (Bio-Rad, Marnes la Coquette, France). The reaction mixture contained 10 μL TB Green Premix Ex Taq II (Tli RNaseH Plus, Takara Bio), 0.8 μL 10 mM primer, 2 μL of a 20-, 40-, 80-, 160-fold dilution series of cDNA, and 6.4 μL water. qRT-PCR was performed using the following protocol: denaturation at 95 °C for 30 s; denaturation and amplification at 95 °C for 5 s and 60 °C for 30 s, respectively (40 cycles). Fluorescence was measured at the end of the amplification step, and amplified products were examined by melting curve analysis from 60 to 95 °C. Each reaction was performed in three independent cultivations. Relative gene expression fold change was calculated using the comparative Ct method (2^−ΔΔCt^) [49]. Normalized expressions were used for reaction in dark. The 2^−ΔΔCt^ values ≤0.5 were defined as downregulated and values ≥2.0 as upregulated, with *P* < 0.01 by the Student’s *t*-test between reactions in light and dark for each gene.

### Chemotaxonomic analysis and other tests

Gram staining was conducted using a modified Hucker method [50]. To study the chemotaxonomic characteristics of WC8-2, we used the cells grown in 20% R2A (pH6.0) with air/CO_2_ (90:10, v/v) at 30 °C for 18 days under a 12/12 h light/dark regimen. Major menaquinone, polar lipids, and cellular fatty acids were determined following established protocols [4]. Briefly, the major menaquinone was isolated by thin-layer chromatography (TLC) [46] and identified using liquid chromatography. Polar lipids were identified using 2D TLC sprayed with detection reagents [51]. The fatty acid composition was identified using the Sherlock Microbial Identification System (v 6.0; MIDI) with the TSBA6 database. These chemotaxonomic analyses were conducted at Techno Suruga Laboratory Co., Ltd. (Japan).

## Results

### “*Ca*. Eremiobacterota” distribution in the fumarolic ice caves on Mt. Erebus

To gain a preliminary understanding of the bacterial communities in the fumarolic ice caves, 16 S rRNA gene amplicon sequencing was performed. Briefly, 273,068 reads were detected and 511 OTUs. The most relatively abundant groups among the samples (>10% on at least one sample) with 97% sequence similarity were *Actinomycetota* (relative abundance: 11.4–54.2%), *Proteobacteria* (6.6–49.2%), *Chloroflexota* (mostly the class *Ktedonobacteria*) (0.5–48.5%), “*Ca*. *Eremiobacterota*” (0.6–17.4%), *Bacteroidota* (0.0–13.8%) and *Gemmatimonadota* (0.2–12.2%) (Fig. S1A and Table S5A). Consistently, cyanobacterial groups were only present in low abundance or nearly absent across all the samples. Focusing on “*Ca*. Eremiobacterota”, Harry’s Dream (HD3) and Mammoth Cave (MM) had a high relative abundance (17.4 and 13.7%, respectively), followed by Haggis Hole (HH) 6.2%, Warren Cave (WC8) 5.6%, Warren Cave (WC7) 1.7%, Hut Cave 1.2%, and Heroine Cave (HC) 0.6% (Table S5A). Diverse OTUs within “*Ca*. Eremiobacterota” were distributed across the samples, with 87.6% of the candidate family “Baltobacteraceae”, 11.3% of the candidate family “Eremiobacteraceae”, and 1.1% of the candidate family “Xenobiaceae” (Fig. S1B, Table S5B). #OTU310 from Warren Cave (WC8), which matched strain WC8-2 with 100% 16 S rRNA partial sequence similarity, had a relative abundance of only 0.1% of the total OTUs (Fig. S1B, Table S5B).

### Isolating the first cultured “*Ca*. Eremiobacterota” strain

We spread nine fumarolic ice cave sediments (WC1, 2, 3, 4, 7, 8, 10, and HD2, 3) previously sampled from Mt. Erebus [21] on solid isolation media and incubated the plates for four weeks. Forty-eight slow-growing colonies were selected and identified by direct colony PCR based on 16S-partial sequences. Twenty-four colonies belonged to the phylum *Firmicutes*, 14 to *Actinomycetota*, six to *Proteobacteria*, one to Chloroflexota, one to *Deinococcota*, and, notably, one to “*Ca*. Eremiobacterota” (Table S6). The eremiobacterial colony was a tiny red colony (<0.5 mm) that appeared after 60 days of incubation of Warren Cave (WC8) samples on 10% R2AG (pH 4.5) without sodium azide (Table S6). This tiny colony was nearly invisible to the naked eye and found using a magnifying glass in a crack in the plate created by directly spreading a sample.

### Phylogeny of strain WC8-2

To obtain further insights into WC8-2’s phylogeny, we isolated a pure culture strain from the colony and sequenced its genome represented by one 3,508,363 bp circular chromosome comprising 68.4 mol% G + C content (Fig. S2). Genome annotation revealed that it comprised 3577 predicted genes, including 57 RNA genes (three rRNA genes and 54 tRNA genes).

**Fig. 1:**
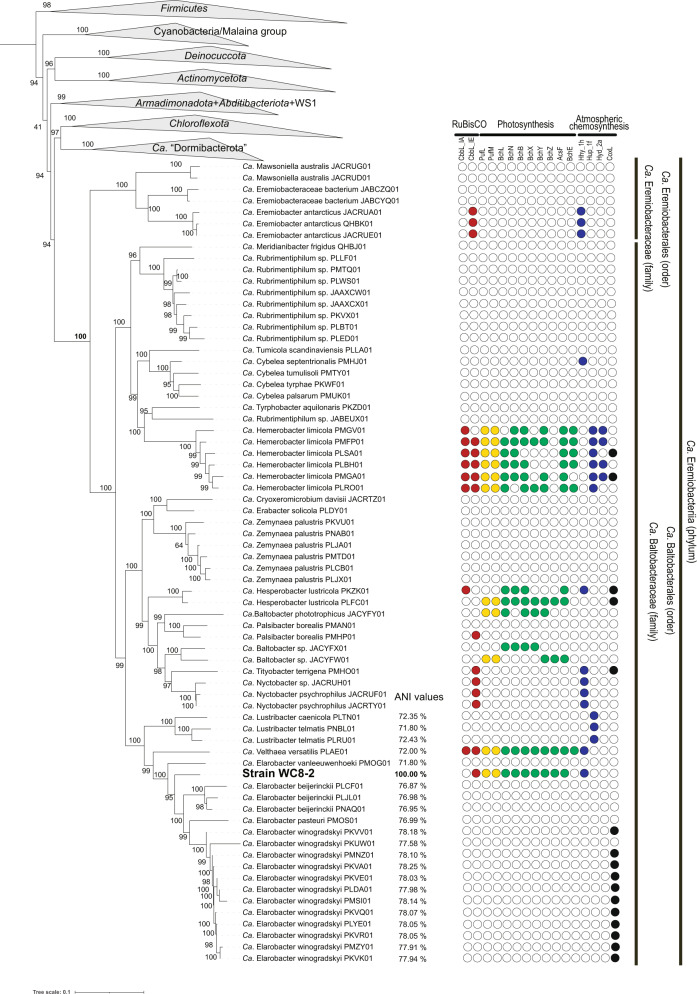
Phylogenomic tree of strain WC8-2 within the Terrabacteria group based on 400 marker protein sequences, with the presences of genes encoding specific proteins involved in carbon fixation, phototrophy, and “atmospheric chemosynthesis” tabulated. The phylogenetic tree was reconstructed using IQ-tree to produce maximum-likelihood trees with 1,000 bootstraps. Bootstrap support is shown as a solid black circle for nodes with greater than 80% support. The presence of the following genes is indicated with a color-filled circle: red for RubisCO (CbbLS), yellow for photoreaction center proteins (PufML), green for BChl synthesis protein (BchLNBXYZE, and AcsF), blue for group 1 and 2 [NiFe]-hydrogenases large subunit (group 1h_Hhy, group 1f_Hup, and group 2a_Hyd), and black for carbon monoxide dehydrogenase (CoxL). The scale bar represents substitutions per nucleotide base. The MAGs information used in this tree is shown in Table S3.

The genome-based phylogeny indicated that WC8-2 was located within a monophyletic lineage consisting of Eremiobacteria MAGs with a 100% bootstrap value (Fig. 1). The AAI value between WC8-2 and representatives of its sister lineage, the phylum *Chloroflexota*, was 38.5–41.2% (Table S7). The genome-based tree also showed that WC8-2 was positioned within a clade consisting of the candidate genera “Elarobacter”, “Velthaea” and “Lustribacter” with ANI values 71.8–78.3%, 72.0%, 71.8–72.4%, respectively (Fig. 1). The 16 S rRNA gene-based phylogeny indicated that WC8-2 was positioned within a clade of “*Ca*. Elarobacter”, with the closest relative (“*Ca*. Elarobacter winogradskyi”) having 97.5 % 16 S rRNA gene sequence similarity (Fig. S3).

### Main Metabolic pathways based on the WC8-2 genome

**Fig. 2:**
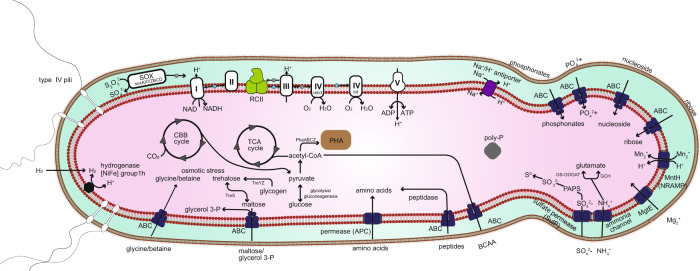
Metabolic reconstruction based on the genome of strain WC8-2. I, NADH dehydrogenase; II, succinate dehydrogenase/fumarate reductase; III, cytochrome bc1 complex; IV cbb3, cbb3-type cytochrome c oxidase; IV bd, cytochrome bd quinol oxidase; V, F-type ATPase; RCII, photoreaction center II; SOX, sulfur/thiosulfate oxidation protein complex; ABC, ABC transporter; APC, amino acid/polyamine/organocation superfamily; NRAMP, natural resistance-associated macrophage proteins; PHA, polyhydroxyalkanoates; Poly-P, polyphosphate granule. Gray and blue circles represent cytochrome c proteins and quinones, respectively. Identification of genes associated with pathways in this overview is given in Supplementary Table S8.

An overview of the metabolic capacity of WC8-2 was constructed based on the genome (Fig. 2 and Table S8). Notably, the genome encodes an anoxygenic Type II RC (pufL, pufM, and pufC), and a complete set of BChl *a* biosynthesis genes (bchL, bchN, bchB, bchX, bchY, and bchZ), whereas bchK, bchU, and bchQ involved in BChl *c*, *d*, and *e* biosynthesis are not encoded. It also encodes the aerobic form of Mg- protoporphyrin IX monomethyl ester oxidative cyclase (acsF), but, like “*Ca*. Hesperobacter” and “*Ca*. Baltobacter” in the putative phototrophic “*Ca*. Eremiobacterota”, it lacks the bchE-encoded anaerobic form [51]. By contrast, “*Ca*. Velthaea” and “*Ca*. Hemerobacter” have both bchE and ascF that may allow phototrophic growth under anaerobic conditions (Fig. 1). WC8-2 has a complete gene set involved in the Calvin–Benson–Basham (CBB) cycle including type IE RuBisCO and phosphoribulokinase (Prk) genes (Fig. 1). No other known autotrophic carbon fixation pathways have been identified in the genome. The WC8-2 genome encodes a group 1 h [NiFe] hydrogenase large subunit (Fig. S4).

The genome has complete gene sets for glycolysis and the citric acid cycle for generating ATP and NAD(P)H (Fig. 2 and Table S8). The genome also has cbb3-type cytochrome c oxidase and cytochrome bd quinol oxidase genes for the respiratory complexes I–IV (Fig. S2 and Table S6).

### Phylogenies of the phototrophy-related proteins

**Fig. 3:**
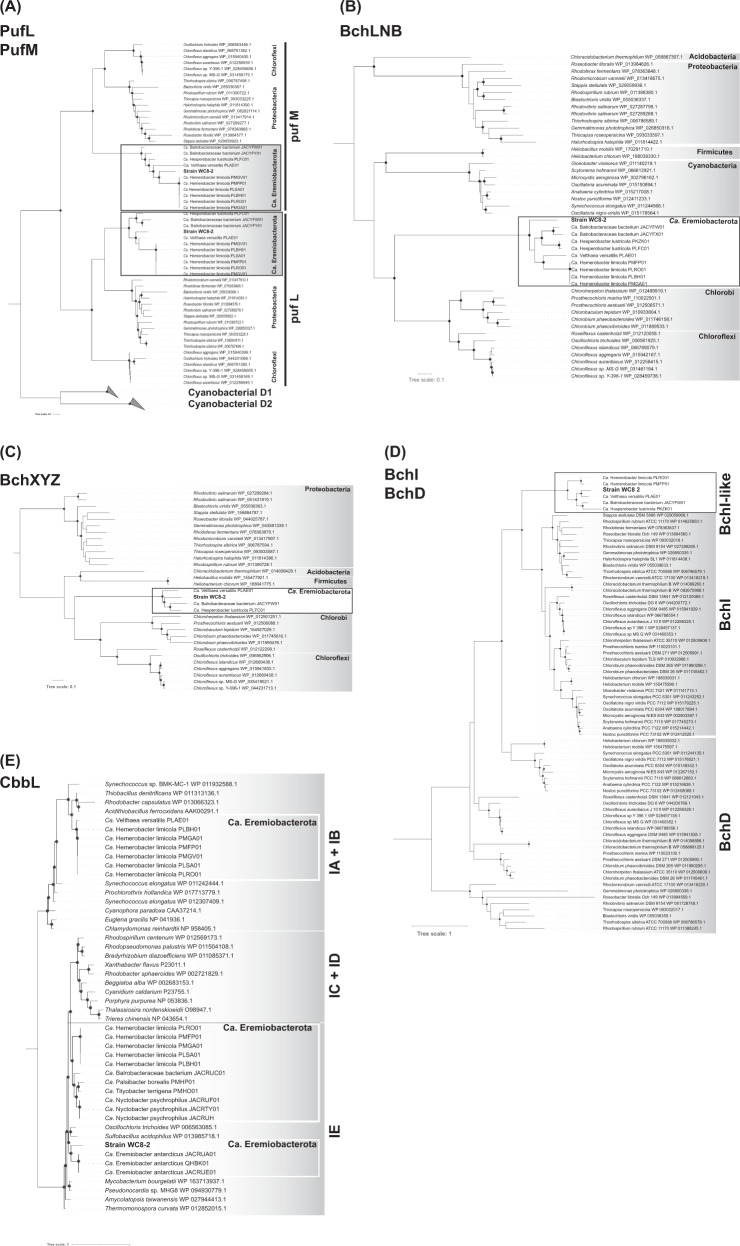
Phylogeny of WC8-2 phototrophy and RubisCO proteins. **A** Maximum likelihood phylogeny of Type II reaction center proteins (374 aligned positions). The phylogenetic tree was reconstructed using IQ-tree with 1000 bootstraps. Bootstrap support is shown as a solid black circle for nodes with greater than 80% support. The phylogeny is midpoint rooted. The scale bar represents the number of amino acid substitutions per site. **B** Maximum likelihood phylogeny of BchLNB concatenated proteins (1160 aligned positions). The phylogenetic tree was reconstructed using IQ-tree with 1000 bootstraps. Bootstrap support is shown as a solid black circle for nodes with greater than 80% support. The phylogeny is midpoint rooted. The scale bar represents the number of amino acid substitutions per site. **C** Maximum likelihood phylogeny of BchXYZ concatenated proteins (1117 aligned positions). The phylogenetic tree was reconstructed using IQ-tree with 1000 bootstraps. Bootstrap support is shown as a solid black circle for nodes with greater than 80% support. The phylogeny is midpoint rooted. The scale bar represents the number of amino acid substitutions per site. **D** Maximum likelihood phylogeny of BchD and BchI proteins (264 aligned positions). The phylogenetic tree was reconstructed using IQ-tree with 1000 bootstraps. Bootstrap support is shown as a solid black circle for nodes with greater than 80% support. The phylogeny is midpoint rooted. The scale bar represents the number of amino acid substitutions per site. **E** Maximum likelihood phylogeny of form I RubisCO large subunit (369 aligned positions). The phylogenetic tree was reconstructed using IQ-tree with 1000 bootstraps. Bootstrap support is shown as a solid black circle for nodes with greater than 80% support. The phylogeny is midpoint rooted. The scale bar represents the number of amino acid substitutions per site.

The phylogenic positions and locations of phototrophy-related proteins in WC8-2 genome were investigated. The protein sequences encoding the RC (PufL and PufM), light-independent protochlorophyllide reductase (BchNLB), and chlorophyllide oxidoreductase (BchXYZ) of the WC8-2 and “*Ca*. Eremiobacterota,” each form a monophyletic clade with deep branching, and are by far the most distant from those of known phototrophs [52] (Fig. 3A, B, C). BchIDH (the bacterial Mg-chelatase complex) catalyzes the first step in chlorophyll or BChl biosynthesis, ATP-dependently inserting Mg_2_ into protoporphyrin IX [51]. The BchID-fused protein formed a monophyletic clade, as did BchLNB and BchXYZ (Fig. 3D).

### Growth characteristics

The growth characteristics of WC8-2 are shown in Fig. S5, Table S9 and summarized in Table 1. WC8-2 grew in low-nutrient media with 1–20% R2A broth or Basal_YE at a temperature range of 13–33 °C and a pH range of 4.5–8.0 (optimal conditions were Basal_YE, 30–33 °C and pH 6.0), and tolerated 0.1% (w/v) NaCl. No growth was observed in full strength R2A, suggesting that it prefers lower nutrient conditions. WC8-2 grew well under aerobic conditions with 12/12 h light/dark regime with N_2_/O_2_/CO_2_ (70:20:10%), moderately under reduced O_2_ conditions with N_2_/O_2_/CO_2_ (80:10:10%), and slowly under microaerophilic conditions with N_2_/O_2_/CO_2_ (89:1:10%). The final OD_600_ value indicating microaerobic growth at N_2_/O_2_/CO_2_ (89:1:10%) was approximately 1/6th the growth at N_2_/O_2_/CO_2_ (70:20:10%) and established that growth was aerobic (Fig. S5). No growth was observed in the absence of O_2_ under any condition tested. It also did not grow on the surface in solid 10% R2AG under atmospheric conditions but grew in stab cultures with tiny red to pink colonies. Despite being aerobic, WC8-2 did not grow in a BIO-SILICO N-38 sponge plugged culture in the presence of air (100%) or in the absence of CO_2_ (N_2_/O_2_/CO_2_ [80:20:0%]) under a 12/12 h light/dark regime, demonstrating that it requires a high CO_2_ concentration for growth even in heterotrophic mode (Fig. S5). WC8-2 showed no growth under anaerobic/fermentative conditions with any electron acceptors tested (Table S9).

**Table 1:** Summary of the phenotypic traits of strain WC8-2.

**Characteristic**	
Cell shape	Short to long rod or filament
Cell size	
Width (μm)	0.5–0.7
Length (μm)	1.0–92.0
Motility	Positive phototaxis
Gram-stain	Negative
Genome size (Mbp)	3.50
DNA G + C content (mol%)	68.4
Temp. for growth ( °C)	
Range	13–33 (no growth at 10 and 37)
Optimum	30–33
pH for growth	
Range	4.5– 8.0 (no growth at 3.7 and 9.0)
Optimum	6.0
NaCl concentration for growth (% w/v)	0–0.1
O_2_ requirement	Aerobe
CO_2_ requirement	High-CO_2_ requirement
Lifestyle	Mixotroph;
Photoheterotrophic growth	+
Chemoheterotrophic growth	+
Photolithoautotrophic growth	−
Chemolithoautotrophic growth	−
Anaerobic/Fermentative growth	−
CO_2_-fixation	+
Pigment	Bacteriochlorophyll *a*
Carbon source utilization pattern	
Sucrose	−
d-Glucose	−
d-Ribose	−
Maltose	−
Glycerol	−
l-Leucine	−
l-Isoleucine	−
l-Valine	−
l-Serine	−
l-Lysine	−
Taurine	−
Yeast extract	+
Gellan gum	−
Major cellular fatty acids % (>10%)	iso-C14:0 (33.9)
	iso-C14:0-3OH (16.5)
	iso-C13:0-3OH (11.0)
Major quinone	MK-7

+ substrate utilized/present, − substrate not utilized/absent

### Phototrophic growth

**Fig. 4:**
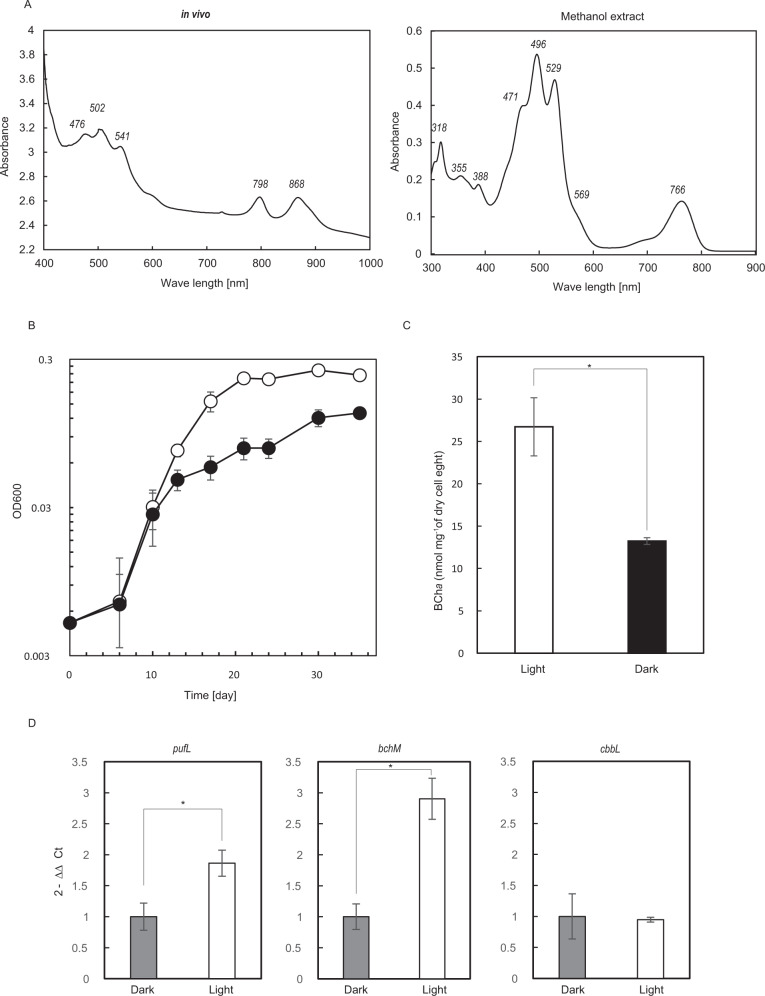
Phototrophic traits of strain WC8-2. **A** Absorption spectra of the pigments. **B** The growth curve under light and dark conditions. The open circles indicate the light conditions, the closed circles for the dark conditions **C** The BChl *a* production under light and dark conditions. **D** Relative transcriptional levels of pufL, bchM, and cbbL under light and dark conditions. Values in B-D indicate the average of three (**A**, **C** and **D**) or six (**B**) independent cultivations. Error bars show the standard deviation of values from three independent cultivations. ^*^P < 0.01.

WC8-2 exhibited no evident photolithoautotrophic or chemolithoautotrophic growth, aerobically or anaerobically, in mineral media (amended PBS2 and Basal media) with any of the electron donors tested (Table S9). In contrast, growth was clearly stimulated by light in heterotrophic and aerobic conditions in Basal_YE. WC8-2 utilized yeast extract (Basal_YE), but was not able to utilize d-glucose, d-ribose, maltose, l-leucine, l-isoleucine, l-valine, l-serine, l-lysine, taurine, and gellan gum under either aerobic photoorganoheterotrophic or aerobic chemoorganoheterotrophic conditions (Fig. 4B, Fig. S5 and Table S9). No growth occurred in any of the anaerobic conditions tested.

### Pigment

Strain WC8-2 produced BChl *a* identified by its absorption spectrum with infrared peaks of 798 and 868 nm *in vivo*, and typical BChl spectral peaks of 355, 569, and 766 nm in a 100% methanol extract (Fig. 4A) and the single HPLC-separated peak (Fig. S6). This result is supported by the presence of a complete gene set for BChl *a* biosynthesis in the genome. Most light absorption between 400 and 600 nm was carotenoid-mediated, with major *in vivo* absorption peaks at 476, 502, and 541 nm (Fig. 4A), similar to that of *Gemmatimonas phototrophica* [13].

### Bacteriochlorophyll-production

Heterotrophic growth was significantly enhanced by illumination in Basal_YE; doubling times were 17 and 21 h in light and dark, respectively (Fig. 4B). In addition, bacteriochlorophyll (BCh) production was also promoted by illumination (*n* = 3, *P* < 0.01); 26.7 ± 3.4 and 13.2 ± 0.4 (nmol^−^^1^ mg dry cell weight) in light and dark, respectively (Fig. 4C).

### CO_2_-fixation

To investigate CO_2_ fixation, growth tests were conducted in the presence of isotope-labeled ^13^CO_2_. The C isotope ratio (δ^13^C) of the WC8-2 cells in the exponential growth phase grown in Basal_YE with non-labeled CO_2_ was −22.9 ± 0.7‰ and −21.7 ± 0.2‰ in light (photoheterotrophic condition) and dark (chemoheterotrophic condition), respectively. That of WC-8 cells with isotope-labeled ^13^CO_2_ was 2279.4 ± 719.9‰ and 1602.2 ± 572.8‰ in the light and dark respectively (Table 2).

**Table 2:** Carbon isotope values of strain WC8-2 cells in growth experiments using labeled ^13^CO_2_ versus unlabeled CO_2_.

Growth condition		Mean δ13C value (%_0_) (*n* = 2)
Unlabeled CO_2_	Light	−22.9 ± 0.7
	Dark	−21.7 ± 0.2
Labeled ^13^CO_2_	Light	2279.4 ± 719.9
	Dark	1602.2 ± 572.8

Strain WC8-2 was cultivated in liquid Basal_YE under a 24 h light or 24 h dark with 10% unlabeled CO_2_ or labeled ^13^CO_2_. Mean values of two independent experiments are shown with standard deviations.

### Gene transcripts related to phototrophy

Transcription of the RC genes (pufL and bchM), the type IE RuBisCO large subunit gene (cbbL), and an internal reference gene (rpoB) was assessed by qRT-PCR using exponentially growing cells. The standard RT-PCR products were identified as the sequences of *pufL*, *bchM*, *cbbL*, and *rpoD* from the WC8-2 genome. The average PCR efficiencies were similar: 97% (light) and 95% (dark) for *pufL*, 95% (light) and 97% (dark) for *bchM*, 99% (light) and 98% (dark) for *cbbL*, and 97% (light) and 98% (dark) for *rpoB* with high correlation coefficients (R^2^ > 99%). In addition, the melting curve analysis confirmed that no primer-dimers were generated during the applied 40 real-time PCR amplification cycles (data not shown).

Figure 4D and Table S10 shows gene transcription levels in light relative to those in dark. The relative bchM transcription level (2^−ΔΔCt^) under light was 2.90 ± 0.33 (*n* = 3, *P* < 0.05). This indicates that illumination upregulates bchM. The pufL transcription level was 1.86 ± 0.21 (*n* = 3, *P* < 0.05). Although this result suggests pufL expression is upregulated by illumination, since the value was less than the 2.0 threshold, which is defined as the lower limit for upregulation, we can not conclude that this increased expression is significant.

### Phototactic behavior

WC8-2 cells grown at the bottom of a culture flask exhibited consistent lateral taxis toward the incandescent light source, suggesting positive phototactic behavior. Macroscopic phototactic responses to various wavelengths of LED light revealed that the cells display positive phototaxis to blue (λP 470 nm), green (λP 527 nm), and red (λP 624 nm) light, possibly by gliding along the bottom of the culture flask. Cells were unresponsive to violet (λP 395 nm) and near-infrared (λP 880 nm) (Table 3 and Movies S1 and S2). Phototaxis was observed at 4–30 °C (Table 3).

**Table 3:** Phototactic behavior of strain WC8-2.

Phototaxis	
λP (nm)	Tactic activity
395 (violet)	Non tactic
470 (blue)	Positive
527 (green)	Positive
624 (red)	Positive
880 (near infrared)	Non tactic
***Temperature (°C) at*** **470, 527 and 624 nm**	
1	Non tactic
4	Positive
10	Positive
20	Positive
30	Positive

### Cell structures

**Fig. 5:**
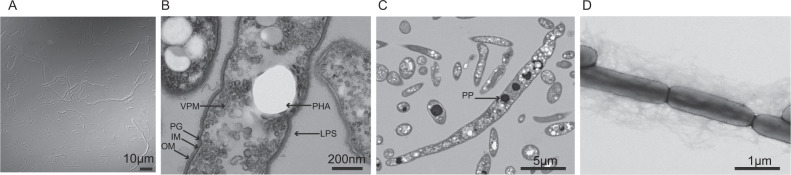
Phase-contrast and transmission electron micrographs of strain WC8-2. **A** Phase-contrast micrograph, scale bar; 10 μm, **B** and **C** Ultrathin section images of the cells showing the cell structure (scale bars; 200 nm for **B**, 5 μm for **C**). **D** Negative stain micrographs (scale bar; 1 μm). The arrows indicate the cell structures: IM Inner membrane, LPS Lipopolysaccharide, MV Membrane vesicle, OM Outer membrane, PG Peptidoglycan, PP polyphosphate granules, PHA Polyhydroxyalkanoates, UG Unidentified granules, VPM Vesicular photosynthetic membranes.

WC8-2 cells are 0.5–0.7 μm diameter and 1.5–92.0 μm long rods or filaments (Fig. 5A). Ultrathin cellular sections revealed a typical gram-negative cell wall comprising an inner cytoplasmic membrane, thin peptidoglycan, and a lipopolysaccharide-covered outer membrane (Fig. 5B, C). A vesicular intracytoplasmic membrane system was observed (Fig. 5B). Since anoxygenic phototrophs generally form photosynthetic membrane systems such as vesicles, tubes, and lamellae, this vesicular membrane may be a photosynthetic membrane system. Negative staining (Fig. 5D), supported by the presence of a gene set encoding type IV pili (pilMDTC) (Table S8) showed that the cells formed type IV pili. No flagella were observed, in agreement with the lack of a flagellin gene (Table S8). The cells produced opaque intracellular inclusions with intense fluorescence after staining with Nile-Red (Fig. S7A, B) known to stain polyhydroxyalkanoate (PHA) [53] and the genome encodes a gene set for PHA synthesis (Table S8). PHA is a well-known system for storing carbon or energy sources under nutrient starvation and plays an essential role in regulating photoheterotrophy in aerobic anoxygenic phototrophs (AAnPs) [54]. Dark-stained granules of varied sizes were observed (Fig. 5C). The granules fluoresced green with DAPI staining, which stains inorganic polyphosphate [55], suggesting that they are polyphosphate granules called acidocalcisomes (Fig. S7A, B). Acidocalcisomes have also been speculated to be involved in acclimation to nutrient deprivation [56].

### Chemotaxonomic feature

As shown in Table1 and Table S11, the major cellular fatty acids were iso-C14:0 (33.9%), iso-C14:0-3OH (16.5%) and iso-C13:0-3OH (11.0%). The major respiratory quinone is menaquinone-9 (MK-9) (Table 1). The polar lipids consisted of an unidentified polar lipid, four unidentified glycolipids, and seven unidentified lipids. The polar lipids profile is shown in Fig. S9.

## Discussion

The members of “*Ca*. Eremiobacterota” are abundant in Antarctic bare soils [7, 18], Arctic permafrost soils [19], boreal mosses [6] and volcanic soils [20], and the MAGs reconstructed from metagenomics include lineages with photosynthetic [5, 6] and atmospheric chemosynthetic potentials [7], suggesting that they are key lineages involved in cycling carbon in “extreme” environments. Despite their ecological importance, no pure cultured “*Ca*. Eremiobacterota” is available, seriously hindering accurate characterization of their metabolism, physiology, cell structure, and ecology.

This study successfully isolated the first “*Ca*. Eremiobacterota” strain in pure culture, strain WC8-2, from a fumarolic ice cave in Antarctica. We investigated its physiology, phylogenetics, cell structure, and genomic traits, revealing that WC8-2 is an AAnP with unique phototrophy-related genes and shows CO_2_ fixation ability, phototaxis, and a high CO_2_-requirement.

Presumed factors allowing the first successful isolation of “*Ca*. Eremiobacterota”, include a low-nutrient medium (10% R2A broth) with gellan gum as a solidifying agent and detecting micro-colonies that appeared inside the cracks under magnification after a long incubation period. The first member of the phylum Abditibacteriota, isolated from Antarctic soil, was similarly observed as a microcolony after 70 days of incubation on a low-nutrient medium [57]. Furthermore, the first members of the phylum Armatimonadetes were isolated using a solid medium solidified with gellan gum [24] which has also been used as a solidifying agent to efficiently isolate novel taxa.

A monophyletic clade of “*Ca* Eremiobacterota” containing WC8-2 is clearly distinct from any other phyla with 100% bootstrap probabilities, which is consistent with the results described previously [6, 7]. As shown in Table S12, members of the Eremiobacterota show less than 80% pairwise 16 S rRNA gene sequence similarities to those of sister cultured phyla (*Chloroflexota* and *Armatimonadota*), falling below the median phylum-level 16 S rRNA gene similarity threshold of 83.68% (range 81.6–85.93%) [58]. Furthermore, as shown in Table S7, WC8-2 has average amino acid identities (AAI) values less than 41.2% with sister cultured phyla, also falling below the median phylum-level AAI threshold of 46.2% [17]. These analyses reconfirm that “*Ca*. Eremiobacterota” is an independent phylum level lineage in the bacterial domain, affiliated with the Terrabacteria superphylum.

As shown in the phylogenetic tree based on 16 S rRNA gene sequences (Fig. S3), WC8-2 falls within a clade of the candidate genus “Elarobacter” with “*Ca*. Elarobacter winogradskyi” as the closest relative (97.5% 16 S rRNA gene sequence similarity). The genome-based phylogeny also showed that WC8-2 falls within a clade of “*Ca*. Elarobacter” with a range of 71.8–78.3% average nucleotide identity (ANI) (Fig. 1). Furthermore, the GTDBtk-based identification analysis also assigned the WC8-2 to “*Ca*. Elarobacter”. However, “*Ca*. Elarobacter” is inferred to be an obligate heterotrophic group with no genetic potential for photosynthesis, CO_2_-fixation, or hydrogen oxidation according to its MAG-based predicted metabolism [4], which is markedly different from the metabolic potentials of WC8-2. The characteristics are rather similar to the candidate genus “Velthaea” with a versatile metabolic system (Fig. 1). Nevertheless, WC8-2 has an ANI value of 72.00% with “*Ca*. Velthaea versatilis” (only one species within the genus is in the GTDB at this time), which is below a median genus-level threshold of a 73.11% [59], and its phylogenomic position is closer to “*Ca*. Elarobacter” than to “*Ca*. Velthaea” (Fig. 1), suggesting that WC8-2 is distinct from “Velthaea” at the genus level. Collectively, it is reasonable to place this strain in a new genus within the candidate family “Baltobacteraceae”.

WC8-2 is a mesophilic (13–33 °C) strict aerobe that can heterotrophically grow in a 1–20% oxygen atmosphere, but prefers a capnophilic lifestyle (Fig. S5). Growth is clearly promoted with increasing O_2_ concentrations in the gas phase, but growth continues to require high CO_2_ concentration (Fig. S5). Growth in a wide range of oxygen concentrations may be enabled by cbb3-type cytochrome c oxidase and cytochrome bd quinol oxidase genes for the respiratory complexes I–IV associated with aerobic respiration under O_2_-limited conditions [60]. The WC8-2 genome encodes many branched-chain amino acids (e.g., leucine, isoleucine, valine) and transporters (LivKHGMF) (Fig. 2 and Table S8), suggesting it possesses a well-developed metabolic system that utilizes extracellular branched-chain amino acids. However, WC8-2 was not able to utilize these branched-chain amino acids as sole carbon sources for growth (Fig. S5). These phenotypes endow WC8-2 with a high adaptability to fumarolic environments given the ubiquitous existence of warm temperatures (15 °C, WC8), a high CO_2_ concentration (~2.9% v/v) and 0.1–23.8% O_2_ in Warren Cave [61].

The WC8-2 genome encodes an anoxygenic Type II RC, a complete set of BChl *a* biosynthesis genes, and a complete set of genes for the CBB cycle (type IE RuBisCO). For potential oxidative energy to drive the CBB cycle, WC8-2, as well as members of “*Ca*. Eremiobacter,” “*Ca*. Tityobacter,” and “*Ca*. Nyctobacter,” possess HhyL-encoding genes, suggesting that members have the potential for “atmospheric chemosynthesis” [4]. Diverse members of “*Ca*. Eremiobacterota” MAGs also possess coxL, the gene encoding one of the subunits of aerobic carbon monoxide dehydrogenase involved in oxidizing atmospheric CO [7, 62] (Fig. 1). Although WC8-2 possesses CoxL homologs (WPS_11190 and WPS_30380), the active site, CSFR [63], is not found in the sequences. Moreover, the WC8-2 genome encodes a complete Sox system involved in thiosulfate oxidation (Fig. 2 and Table S8). Despite such genetic potential, no photolithoautotrophic or chemolithoautotrophic growth was observed using H_2_, sodium thiosulfate, or sodium sulfide as electron donors. It remains possible that the conditions for lithoautotrophic growth were just not found in this study.

Importantly, WC8-2 produces bacteriochlorophyll *a* and illumination markedly enhanced its growth and BChl *a* production under heterotrophic conditions using Basal_YE (Fig. 4C). Furthermore, light upregulates BCh synthesis-associated bchM expression in WC8-2 during exponential growth in Basal_YE (Fig. 4D). Such features clearly indicate that this bacterium is a BChl *a*-based photoheterotroph, but the electron donor could not be identified in this study. On the other hand, the C isotope ratios (δ^13^C) of WC8-2 cells grown in Basal_YE under light or dark conditions with isotope-labeled ^13^CO_2_ were 2279.4 ± 719.9‰ and 1602.2 ± 572.8‰, respectively, with no significant difference, but significantly higher than that of non-labeled WC8-2 cells (Table 2). These values are comparable to δ^13^C values 1540–1677‰ for CO_2_-fixation via 3-hydroxypropionate/4-hydroxybutyrate in the bacterium *Metallosphaera yellowstonensis* grown with ^13^CO_2_ in the presence of yeast extract, but not nearly as high as δ^13^C values of the cells (71,500–80,637‰) grown in the absence of yeast with ^13^CO_2_ [64]. This result suggests that WC8-2 may be able to utilize low levels of inorganic carbon under heterotrophic conditions. Interestingly, WC8-2 expressed *cbbL* during exponential growth in Basal_YE under both light and dark conditions, but there was no significant difference in relative expression, suggesting that the CBB cycle is not driven by light under heterotrophic conditions. This CO_2_-fixation could be driven by trace hydrogen molecules or sulfur compounds, but given there was no autotrophic growth in the mineral medium without organic carbon and WC8-2 possesses the phosphoenolpyruvate carboxylase gene (WPS_26910), it is more likely CO_2_ fixation occurs via anaplerotic pathways to replenish the citric acid-cycle under nutrient-poor conditions rather than the CBB cycle [65]. The expression of *cbbL* is unexplained but may not be functional; further molecular enzymatic studies are needed to identify the CO_2_-fixation pathways. Taken together, WC8-2 is a unique photoheterotroph and a chemoheterotroph with a potential for CO_2_-fixation; we speculate that light energy did not drive the CO_2_-fixation but was used as an auxiliary energy source to activate metabolism, thereby stimulating growth under the light conditions.

Phototaxis allows WC8-2 to seek optimal conditions for its lifestyle through gliding motility with type IV pili on solid surfaces. Interestingly, it is phototactic towards blue, green, and red light (γP 470, 527, and 624 nm). Diverse bacteria such as cyanobacteria, purple bacteria, and haloarchaea are also phototactic, but lineages that are phototactic in blue light are rare due to avoidance of UV light [66]. The WC8-2 genome contains genes homologous to a photoactive yellow protein (WPS_26150) and rhodopsin (WPS_12390), both known as photoreceptor genes; however, the functions of the WC8-2 genes have not been identified. Surprisingly, this strain showed positive phototaxis over 4–30 °C, even though it did not grow below 10 °C. All of these properties may be adaptations to using snow-filtered sunlight in polar and alpine areas, as the wavelengths that pass through snow with maximum transmittance are between 450 and 550 nm [67]. In addition, they could offer a survival advantage in polar volcanic regions, where sunlight and temperature vary dramatically, moving on rock surfaces to seek light.

The question arises: why did this “*Ca*. Eremiobacterota” lineage acquire photosynthetic functions during evolution, even though its habitat, Warren Cave, is completely dark? Although light may be an important energy source for some “*Ca*. Eremiobacterota” lineages, it is clearly not important to all (Fig. 1). This may explain why there is no clear pattern in the apparent abundance of “*Ca*. Eremiobacterota” associated with the light regime of the caves (Fig. S1A, Table S1 and S5). For example, Harry’s Dream is exposed to seasonal light and has a relatively high abundance of 16 S rRNA gene amplicon reads, but other caves with similar seasonal light regimes (Haggis Hole, Hut Cave and Heroine Cave) had a lower abundance. Conversely, Mammoth Cave, which is dark, had a relatively high abundance of “*Ca*. Eremiobacterota” sequences. It is likely that traits other than photosynthesis, such as the ability to gain energy from inorganic sources (e.g., H_2_ or sulfur compounds) and a dependence on high CO_2_ atmospheres are what determines the growth and survival of “*Ca*. Eremiobacterota” in the caves.

In addition to the environmental conditions that control the growth of WC8-2-like organisms in situ, we have to consider where they may have come from. The caves on Mt. Erebus form a dynamic network with possible interconnections between them that open and close depending on the volcano’s activity. Additionally, the volcano periodically spews volcanic rocks and ash that fall onto the surface of the snow and ice. This material becomes buried by additional snow and ice and as the ice melts from inside the caves it eventually is deposited to the sediments and soils found in the caves. Any microorganisms attached to this material will likely have been exposed to seasonal sunlight at some time in their history which may also explain why some organisms in the dark caves have photosynthetic abilities.

WC8-2 cells contain acidocalcisomes and PHA (Fig. S8), which are involved in energy storage and starvation acclimation, respectively [54, 56], and its genome encodes trehalose biosynthetic and betaine/glycine transporter genes (Fig. 2 and Table S8), which are involved in adaptation to low-temperature environments [68, 69]. These features may help WC8-2 survive in the polar, oligotrophic and fumarolic environments of Mt. Erebus where slight differences in position can cause steep gradients in energy source (chemical and light) and temperature [70].

WC8-2 has uncharted Type II RC (PufL and PufM) and BChl synthesis proteins that form a deeply branched monophyletic clade with other putative phototrophic “*Ca*. Eremiobacterota” and distinct from known phototrophs (Fig. 3). The phototrophy-related genes were likely transferred and scattered within the phylum Eremiobacterota, expanding the phototrophic lineages within this phylum [5]. As the directionality of transfers and the relationships among the deep branches remain ambiguous in many cases, the origin and history of the photosynthetic evolution of “*Ca*. Eremiobacterota” is difficult to conclude with current data alone.

Interestingly, the WC8-2 genome and “*Ca*. Eremiobacterota” MAGs possibly encode a BchI and BchD fused protein. The BchID-fused protein contains ATP-binding Walker A and Walker B motifs for BchI, and a C-terminal Von Willebrand factor type A (vWA) domain [71] for BchD. Generally, phototrophy-related genes in Proteobacteria are positioned in a coherent group in the genome as a “photosynthetic gene cluster” (PGC) [72]. The phototrophy-related genes of WC8-2 are more widely distributed throughout its genome than those of Proteobacteria, with non-photosynthetic genes mixed in with the WC8-2 PGCs, but they are relatively grouped without the Mb-class long inserts found in the Chloroflexota and Chlorobiota PGCs (Fig. S2) [73]. The WC8-2 PGC contained gene sub-clusters bchM-acsF, bchNBGP, bchFCXYZ, and puf operon (puf BALMC). The gene arrangement bchM-acsF in WC8-2 is also found in Chlorobiota [73], Firmicutes [13], and “*Ca*. Eremiobacterota” MAGs (“Velthaea” and “Hemerobacter”) [5], and the operon pufBALMC is one of the common sub-clusters in phototrophic Proteobacteria [74]. Sub-clusters bchNBGP and bchFCXYZ are also found in “*Ca*. Eremiobacterota” MAGs (“Velthaea” and “Hesperobacter”) [5] and located downstream of bchL with several non-photosynthetic genes. Characteristically, a gene encoding a RuBisCo-like protein (type IV_deep cluster) is embedded between sub-clusters bchNBGP and bchL. The role of type IV RubisCO-like proteins is still unknown [75]. This arrangement and orientation are unusual features of other anoxygenic phototroph PGCs, but they are somewhat similar to that of *Blastochloris*, where some genes encoding CBB cycle proteins are embedded in the PGC [76]. WC8-2 is the third cultured phototrophic phylum based on Type II RC since its discovery in Chloroflexota about half a century ago [77]. We believe that this AAnP culture in “*Ca*. Eremiobacterota” will enable the identification of the three-dimensional structures and functions of the phototrophy-related proteins.

Our research has shown that the first cultured “*Ca*. Eremiobacterota” strain WC8-2 is metabolically versatile, capable of growing both photoheterotrophically and chemoheterotrophically and thrives in fumarolic ice caves; produces BChl *a*, PHA, and acidocalcisomes; fixes CO_2_, possibly autotrophically (has the complete CBB cycle); displays high CO_2_ demand, phototaxis with type IV pili, and is adapted to polar and oligotrophic fumarolic environments. The genome encodes novel RC and BChl synthesizing proteins, forming a deeply branched monophyletic clade distinct from known phototrophs. The metabolic versatility displayed by WC8-2 is likely an adaptation strategy that helps it to adjust to rapidly changing conditions in oligotrophic environments. Metabolic versatility appears to be a feature common to organisms thriving in a range of different oligotrophic environments, for example microbes colonizing deep-sea basalt associated with hydrothermal vents and desert environments [78–81].

Based on this polyphasic characterization, we propose strain WC8-2 as a new species and a genus, *Vulcanimicrobium alpinus* gen. nov., sp. nov. Based on the phylogenetic and phylogenomic analyses, we further propose a new phylum *Eremiobacterota* phy. nov.

### Description of *Vulcanimicrobium* gen. nov

*Vulcanimicrobium* (Vul.ca.ni.mi.cro.bi.um. L. masc. n. Vulcanus, the Roman god of fire; N.L. neut. n. microbium, a microbe; from Gr. masc. adj. mikros, small; from Gr. masc. n. bios, life; N.L. neut. n. Vulcanimicrobium, a microbe living in volcanic areas).

Aerobic but a high CO_2_-requirement, Gram-negative, phototaxis, non-spore- forming heterotroph. The major cellular fatty acids are iso-C14:0 (33.9%), iso-C14:0-3OH (16.5%) and iso-C13:0-3OH (11.0%). The major respiratory quinone is menaquinone-9 (MK-9) (Table 1). The polar lipids consisted of an unidentified polar lipid, four unidentified glycolipids, and seven unidentified lipids. DNA G + C content of the type species is 68.4 mol%. The type species is *Vulcanimicrobium alpinus*.

### Description of *Vulcanimicrobium alpinus* sp. nov

*Vulcanimicrobium alpinus* (al.pi’nus. L. masc. adj. alpinus, alpine, referring to the isolation of the type strain from an alpine environment).

Shows the following characteristics in addition to those given for the genus. Cells are a rod or filament with 0.5–0.7 μm diameter and 1.5–92.0 μm long with pili. The colonies are tiny and red-pigmented in stab cultures. Cells grow heterotrophically under aerobic conditions, but do not grow lithotrophically with 5 mM Na_2_S or Na_2_S_2_O_3_ or 1% H_2_ (v/v; in the gas phase) as electron donors under either aerobic or anaerobic conditions with 5 mM Na_2_SO_4_, NaNO_3_, or dimethyl sulfoxide as electron acceptors. Produce bacteriochlorophyll *a*. Grows at 13–33  °C (optimally at 30  °C), at pH 4.5–8.0 (optimally at pH 6.0) and tolerated 0.1% (w/v) NaCl. WC8-2 utilized yeast extract, but was not able to utilize d-glucose, d-ribose, maltose, l-leucine, l-isoleucine, l-valine, l-serine, l-lysine, taurine, and gellan gum. Growth is enhanced under light. The type strain, WC8-2^T^ (=DSM 115291^T^ = BCRC 81314^T^ = NBRC 115847^T^), was isolated from a sediment derived from Warren Cave, fumarolic ice cave, in Mt. Erebus volcano (Antarctica).

### Description of *Baltobacteraceae* fam. nov

Baltobacteraceae (Bal.to.bac.te.ra.ce’ae. N.L. masc. n. *Baltobacter* an original Candidatus generic name; from N.L. fem. n. *balte*, swamp; from N.L. masc. n. *bacter*, rod; -*aceae* ending to denote a family; N.L. fem. pl. n. *Baltobacteraceae* the Baltobacter family).

The description is the same as for the genus *Vulcanimicrobium*. Type genus is *Vulcanimicrobium*.

### Description of *Baltobacterales* ord. nov

Baltobacterales (Bal.to.bac.te.ra’les. N.L. masc. n. *Baltobacter* an original Candidatus generic name; -*ales* ending to denote an order; N.L. fem. pl. n. *Baltobacterales* the Baltobacter order.

The description is the same as for the genus *Vulcanimicrobium*. Type genus is *Vulcanimicrobium*.

### Description of *Eremiobacteriia* class. nov

Eremiobacteriia (E.re.mi.o.bac.te.ri’i.a. N.L. neut. n. *Eremiobacterium*, an original Candidatus genus; from Gr. fem. n. *erêmia*, desert; from N.L. neut. n. *bacterium*, a rod; N.L. neut. pl. n. suff. -*ia*, ending to denote a class; N.L. neut. pl. n. *Eremiobacteriia*, the Eremiobacterium class.

The description is the same as for the genus *Vulcanimicrobium*. Type genus is *Vulcanimicrobium*.

### Description of *Eremiobacterota* phyl. nov

*Eremiobacterota* (E.re.mi.o.bac.ter.ae.o’ta: N.L. masc. n. *Eremiobacter*, an original Candidatus genus; suff. -*aeota* ending denoting a phylum; N.L. pl. neut. n. *Eremiobacterota* the Eremiobacter phylum).

The description is the same as for the genus *Vulcanimicrobium*. Type genus is *Vulcanimicrobium*.

### Supplementary information

Supplementary Figures

Supplementary Tables

Supplementary Table S2

Supplementary Table S3

Supplementary Table S8

Supplementary Table S12

Movie S1

Movie S2

## Data Availability

Pure cultured strain WC8-2 was deposited in the German Collection of Microorganisms and Cell Cultures (DSM 115291^T^), the Bioresource Collection and Research Center (BCRC 81314^T^) and the NITE Biological Resource Center (NBRC 115847^T^). The 16 S rRNA sequence was deposited in the GenBank database (accession number: LC579935). The complete genome sequence has been deposited in GenBank under accession number AP025523. The raw amplicon sequencing data have also been deposited in the DDBJ Sequence Read Archive (accession number: DRA013282).

## References

[1] Bull AT, Asenjo JA, Goodfellow M, Gómez-Silva B (2016). The Atacama Desert: Technical Resources and the Growing Importance of Novel Microbial Diversity. Annu Rev Microbiol.

[2] Vartoukian SR, Palmer RM, Wade WG (2010). Strategies for culture of “unculturable” bacteria. FEMS Microbiol Lett.

[3] Lambrechts S, Willems A, Tahon G (2019). Uncovering the uncultivated majority in antarctic soils: Toward a synergistic approach. Front Microbiol.

[4] Ji M, Williams TJ, Montgomery K, Wong HL, Zaugg J, Berengut JF (2021). *Candidatus* Eremiobacterota, a metabolically and phylogenetically diverse terrestrial phylum with acid-tolerant adaptations. ISME J.

[5] Ward LM, Cardona T, Holland-Moritz H (2019). Evolutionary Implications of Anoxygenic Phototrophy in the Bacterial Phylum *Candidatus* Eremiobacterota (WPS-2). Front Microbiol.

[6] Holland-Moritz H, Stuart J, Lewis LR, Miller S, Mack MC, McDaniel SF (2018). Novel bacterial lineages associated with boreal moss species. Environ Microbiol.

[7] Ji M, Greening C, Vanwonterghem I, Carere CR, Bay SK, Steen JA (2017). Atmospheric trace gases support primary production in Antarctic desert surface soil. Nature.

[8] Ray AE, Zhang E, Terauds A, Ji M, Kong W, Ferrari BC (2020). Soil Microbiomes With the Genetic Capacity for Atmospheric Chemosynthesis Are Widespread Across the Poles and Are Associated With Moisture, Carbon, and Nitrogen Limitation. Front Microbiol.

[9] Gest H, Blankenship RE (2004). Time line of discoveries: Anoxygenic bacterial photosynthesis. Photosynth Res.

[10] Bryant DA, Costas AMG, Maresca JA, Gomez A, Chew M, Heidelberg JF (2007). *Candidatus* Chloracidobacterium thermophilum: An Aerobic Phototrophic Acidobacterium. Science.

[11] Gest H, Favinger JL (1983). *Heliobacterium chlorum*, an anoxygenic brownish-green photosynthetic bacterium containing a “new” form of bacteriochlorophyll. Arch Microbiol.

[12] Pierson BK, Castenholz RW (1974). A phototrophic gliding filamentous bacterium of hot springs, *Chloroflexus aurantiacus*, gen. and sp. nov. Arch Microbiol.

[13] Zeng Y, Feng F, Medová H, Dean J, Koblížek M (2014). Functional type 2 photosynthetic reaction centers found in the rare bacterial phylum Gemmatimonadetes. Proc Natl Acad Sci.

[14] Lewis WH, Tahon G, Geesink P, Sousa DZ, Ettema TJG (2021). Innovations to culturing the uncultured microbial majority. Nat Rev Microbiol.

[15] Nogales B, Moore ERB, Llobet-Brossa E, Rossello-Mora R, Amann R, Timmis KN (2001). Combined Use of 16S Ribosomal DNA and 16S rRNA to Study the Bacterial Community of Polychlorinated Biphenyl-Polluted Soil. Appl Environ Microbiol.

[16] Paoli L, Ruscheweyh H-J, Forneris CC, Kautsar S, Clayssen Q, Salazar G, et al. Biosynthetic potential of the global ocean microbiome. Nature. 2022;111–18. 10.1038/s41586-022-04862-3 PMC9259500 35732736

[17] Parks DH, Rinke C, Chuvochina M, Chaumeil PA, Woodcroft BJ, Evans PN (2017). Recovery of nearly 8,000 metagenome-assembled genomes substantially expands the tree of life. Nat Microbiol.

[18] Ji M, van Dorst J, Bissett A, Brown MV, Palmer AS, Snape I (2016). Microbial diversity at Mitchell Peninsula, Eastern Antarctica: a potential biodiversity “hotspot.”. Polar Biol.

[19] Woodcroft BJ, Singleton CM, Boyd JA, Evans PN, Emerson JB, Zayed AAF (2018). Genome-centric view of carbon processing in thawing permafrost. Nature.

[20] Aszalós JM, Szabó A, Megyes M, Anda D, Nagy B, Borsodi AK (2020). Effects of volcanism on bacterial communities in the crater lake of Ojos Del Salado. Astrobiology.

[21] Tebo BM, Davis RE, Anitori RP, Connell LB, Schiffman P, Staudigel H (2015). Microbial communities in dark oligotrophic volcanic ice cave ecosystems of Mt. Erebus, Antarctica. Front Microbiol.

[22] Schmidt SK, Gendron EMS, Vincent K, Solon AJ, Sommers P, Schubert ZR (2018). Life at extreme elevations on Atacama volcanoes: the closest thing to Mars on Earth?. Antonie van Leeuwenhoek, Int J Gen Mol Microbiol.

[23] Wang C, Zheng Y, Sakai Y, Toyoda A, Minakuchi Y, Abe K (2019). *Tengunoibacter tsumagoiensis* gen.nov., sp. nov., *Dictyobacter kobayashii* sp. nov., *Dictyobacter alpinus* sp. nov., and description of *Dictyobacteraceae* fam. nov. within the order *Ktedonobacterales* isolated from Tengu-no-m. Int J Syst Evol Microbiol.

[24] Stott MB, Crowe MA, Mountain BW, Smirnova AV, Hou S, Alam M (2008). Isolation of novel bacteria, including a candidate division, from geothermal soils in New Zealand. Environ Microbiol.

[25] Zheng Y, Saitou A, Wang CM, Toyoda A, Minakuchi Y, Sekiguchi Y (2019). Genome features and secondary metabolites biosynthetic potential of the class *Ktedonobacteria*. Front Microbiol.

[26] Wick RR, Judd LM, Gorrie CL, Holt KE (2017). Unicycler: Resolving bacterial genome assemblies from short and long sequencing reads. PLoS Comput Biol.

[27] Parks DH, Imelfort M, Skennerton CT, Hugenholtz P, Tyson GW (2015). CheckM: Assessing the quality of microbial genomes recovered from isolates, single cells, and metagenomes. Genome Res.

[28] Tanizawa Y, Fujisawa T, Nakamura Y (2018). DFAST: A flexible prokaryotic genome annotation pipeline for faster genome publication. Bioinformatics.

[29] Grant JR, Stothard P (2008). The CGView Server: a comparative genomics tool for circular genomes. Nucl Acids Res.

[30] Chaumeil PA, Mussig AJ, Hugenholtz P, Parks DH (2020). GTDB-Tk: A toolkit to classify genomes with the genome taxonomy database. Bioinformatics.

[31] Pruesse E, Peplies J, Glöckner FO (2012). SINA: Accurate high-throughput multiple sequence alignment of ribosomal RNA genes. Bioinformatics.

[32] Nguyen LT, Schmidt HA, Von Haeseler A, Minh BQ (2015). IQ-TREE: A fast and effective stochastic algorithm for estimating maximum-likelihood phylogenies. Mol Biol Evol.

[33] Kalyaanamoorthy S, Minh BQ, Wong TKF, Von Haeseler A, Jermiin LS (2017). ModelFinder: Fast model selection for accurate phylogenetic estimates. Nat Methods.

[34] Minh BQ, Nguyen MAT, Von Haeseler A (2013). Ultrafast approximation for phylogenetic bootstrap. Mol Biol Evol.

[35] Asnicar F, Thomas AM, Beghini F, Mengoni C, Manara S, Manghi P (2020). Precise phylogenetic analysis of microbial isolates and genomes from metagenomes using PhyloPhlAn 3.0. Nat Commun.

[36] Buchfink B, Xie C, Huson DH (2014). Fast and sensitive protein alignment using DIAMOND. Nat Methods.

[37] Katoh K, Standley DM (2013). MAFFT multiple sequence alignment software version 7: Improvements in performance and usability. Mol Biol Evol.

[38] Lee I, Kim YO, Park SC, Chun J (2016). OrthoANI: An improved algorithm and software for calculating average nucleotide identity. Int J Syst Evol Microbiol.

[39] Priyam A, Woodcroft BJ, Rai V, Moghul I, Munagala A, Ter F (2019). Sequenceserver: A Modern Graphical User Interface for Custom BLAST Databases. Mol Biol Evol.

[40] Castresana J (2000). Selection of conserved blocks from multiple alignments for their use in phylogenetic analysis. Mol Biol Evol.

[41] Letunic I, Bork P (2021). Interactive tree of life (iTOL) v5: An online tool for phylogenetic tree display and annotation. Nucl Acids Res.

[42] Lopes H, de FS, Tu Z, Sumi H, Yumoto I (2021). Analysis of bacterial flora of indigo fermentation fluids utilizing composted indigo leaves (sukumo) and indigo extracted from plants (Ryukyu-ai and Indian indigo). J Biosci Bioeng.

[43] Zhang J, Kobert K, Flouri T, Stamatakis A (2014). PEAR: A fast and accurate Illumina Paired-End reAd mergeR. Bioinformatics.

[44] Shen W, Le S, Li Y, Hu F (2016). SeqKit: A cross-platform and ultrafast toolkit for FASTA/Q file manipulation. PLoS One.

[45] Saitou N, Nei M (1987). The neighbor-joining method: a new method for reconstructing phylogenetic trees. Mol Biol Evol.

[46] Hiraishi A, Nagao N, Yonekawa C, Umekage S, Kikuchi Y, Eki T (2020). Distribution of Phototrophic Purple Nonsulfur Bacteria in Massive Blooms in Coastal and Wastewater Ditch Environments. Microorganisms.

[47] Permentier HP, Schmidt KA, Kobayashi M, Akiyama M, Hager-Braun C, Neerken S (2000). Composition and optical properties of reaction centre core complexes from the green sulfur bacteria *Prosthecochloris aestuarii* and *Chlorobium tepidum*. Photosynth Res.

[48] Untergasser A, Cutcutache I, Koressaar T, Ye J, Faircloth BC, Remm M (2012). Primer3-new capabilities and interfaces. Nucl Acids Res.

[49] Michael W (2001). Pfaffl. A new mathematical model for relative quantification in real-time RT-PCR. Nucl Acids Res.

[50] Smibert RM, Krieg NR. Phenotypic characterization. In *Methods for General and Molecular Bacteriology*. Gerhardt P, Murray RGE, Wood WA, Krieg NR eds. American Society for Microbiology, Washington, DC, 1994, pp. 607–54.

[51] Chew AGM, Bryant DA (2007). Chlorophyll biosynthesis in bacteria: The origins of structural and functional diversity. Annu Rev Microbiol.

[52] Cardona T (2015). A fresh look at the evolution and diversification of photochemical reaction centers. Photosynth Res.

[53] Spiekermann P, Rehm BHA, Kalscheuer R, Baumeister D, Steinbüchel A (1999). PHA staining Nile red plates. Arch Microbiol.

[54] Xiao N, Jiao N (2011). Formation of polyhydroxyalkanoate in aerobic anoxygenic phototrophic bacteria and its relationship to carbon source and light availability. Appl Environ Microbiol.

[55] Gomes FM, Ramos IB, Wendt C, Girard-Dias W, De Souza W, Machado EA (2013). New insights into the in situ microscopic visualization and quantification of inorganic polyphosphate stores by 4’,6-diamidino-2-phenylindole (DAPI)-staining. Eur J Histochem.

[56] Sanz-Luque E, Bhaya D, Grossman AR (2020). Polyphosphate: A Multifunctional Metabolite in Cyanobacteria and Algae. Front Plant Sci.

[57] Tahon G, Tytgat B, Lebbe L, Carlier A, Willems A (2018). *Abditibacterium utsteinense* sp. nov., the first cultivated member of candidate phylum FBP, isolated from ice-free Antarctic soil samples. Syst Appl Microbiol.

[58] Yarza P, Yilmaz P, Pruesse E, Glöckner FO, Ludwig W, Schleifer KH (2014). Uniting the classification of cultured and uncultured bacteria and archaea using 16S rRNA gene sequences. Nat Rev Microbiol.

[59] Barco RA, Garrity GM, Scott JJ, Amend JP, Nealson KH, Emerson D (2020). A genus definition for bacteria and archaea based on a standard genome relatedness index. MBio.

[60] Borisov VB, Gennis RB, Hemp J, Verkhovsky MI (2011). The cytochrome bd respiratory oxygen reductases. Biochim Biophys Acta - Bioenerg.

[61] Ilanko T, Fischer TP, Kyle P, Curtis A, Lee H, Sano Y (2019). Modification of fumarolic gases by the ice-covered edifice of Erebus volcano Antarctica. J Volcanol Geotherm Res.

[62] Grostern A, Alvarez-Cohen L (2013). RubisCO-based CO_2_ fixation and C1 metabolism in the actinobacterium Pseudonocardia dioxanivorans CB1190. Environ Microbiol.

[63] Dunfield KE, King GM (2004). Molecular analysis of carbon monoxide-oxidizing bacteria associated with recent Hawaiian volcanic deposits. Appl Environ Microbiol.

[64] Jennings RM, Whitmore LM, Moran JJ, Kreuzer HW, Inskeep WP (2014). Carbon dioxide fixation by Metallosphaera yellowstonensis and acidothermophilic iron-oxidizing microbial communities from Yellowstone National Park. Appl Environ Microbiol.

[65] Moran MA, Miller WL (2007). Resourceful heterotrophs make the most of light in the coastal ocean. Nat Rev Microbiol.

[66] Wilde A, Mullineaux CW (2017). Light-controlled motility in prokaryotes and the problem of directional light perception. FEMS Microbiol Rev.

[67] Perovich DK (2007). Light reflection and transmission by a temperate snow cover. J Glaciol.

[68] Kandror O, DeLeon A, Goldberg AL (2002). Trehalose synthesis is induced upon exposure of *Escherichia coli* to cold and is essential for viability at low temperatures. Proc Natl Acad Sci.

[69] Deshnium P, Gombos Z, Nishiyama Y, Murata N (1997). The action in vivo of glycine betaine in enhancement of tolerance of *Synechococcus* sp. strain PCC 7942 to low temperature. J Bacteriol.

[70] Noell SE, Baptista MS, Smith E, McDonald IR, Lee CK, Stott MB (2022). Unique Geothermal Chemistry Shapes Microbial Communities on Mt. Erebus, Antarctica. Front Microbiol.

[71] Lundqvist J, Braumann I, Kurowska M, Müller AH, Hansson M (2013). Catalytic turnover triggers exchange of subunits of the magnesium chelatase AAA+ motor unit. J Biol Chem.

[72] Nagashima S, Nagashima KVP. Comparison of Photosynthesis Gene Clusters Retrieved from Total Genome Sequences of Purple Bacteria. *Adv. Botanical Res*. 2013;66:151–78.

[73] Tang KH, Barry K, Chertkov O, Dalin E, Han CS, Hauser LJ (2011). Complete genome sequence of the filamentous anoxygenic phototrophic bacterium *Chloroflexus aurantiacus*. BMC Genomics.

[74] Zheng Q, Zhang R, Koblížek M, Boldareva EN, Yurkov V, Yan S (2011). Diverse arrangement of photosynthetic gene clusters in aerobic anoxygenic phototrophic bacteria. PLoS One.

[75] Tabita FR, Satagopan S, Hanson TE, Kreel NE, Scott SS (2008). Distinct form I, II, III, and IV Rubisco proteins from the three kingdoms of life provide clues about Rubisco evolution and structure/function relationships. J Exp Bot.

[76] Madigan MT, Resnick SM, Kempher ML, Dohnalkova AC, Takaichi S, Wang-Otomo ZY (2019). Blastochloris tepida, sp. nov., a thermophilic species of the bacteriochlorophyll b-containing genus Blastochloris. Arch Microbiol.

[77] Pierson BK, Castenholz RW (1971). Bacteriochlorophylls in Gliding Filamentous Prokaryotes from Hot Springs. Nat New Biol.

[78] Sudek LA, Templeton AS, Tebo BM, Staudigel H (2009). Microbial Ecology of Fe (hydr)oxide Mats and Basaltic Rock from Vailulu’u Seamount, American Samoa. Geomicrobiol J.

[79] Sudek LA, Wanger G, Templeton AS, Staudigel H, Tebo BM (2017). Submarine basaltic glass colonization by the heterotrophic Fe(II)-oxidizing and siderophore-producing deep-sea bacterium *Pseudomonas stutzeri* VS-10: The potential role of basalt in enhancing growth. Front Microbiol.

[80] Mandakovic D, Cintolesi Á, Maldonado J, Mendoza SN, Aïte M, Gaete A (2020). Genome-scale metabolic models of Microbacterium species isolated from a high altitude desert environment. Sci Rep.

[81] Nakai R, Fujisawa T, Nakamura Y, Baba T, Nishijima M, Karray F (2016). Genome sequence and overview of *Oligoflexus tunisiensis* Shr3T in the eighth class *Oligoflexia* of the phylum *Proteobacteria*. Stand Genomic Sci.

