# Correction: *Vulcanimicrobium alpinus* gen. nov. sp. nov., the first cultivated representative of the candidate phylum “Eremiobacterota”, is a metabolically versatile aerobic anoxygenic phototroph

**DOI:** 10.1038/s43705-023-00301-0

**Published:** 2023-10-18

**Authors:** Shuhei Yabe, Kiyoaki Muto, Keietsu Abe, Akira Yokota, Hubert Staudigel, Bradley M. Tebo

**Affiliations:** 1 Department of Microbial Resources, Graduate School of Agricultural Sciences, Tohoku University, Sendai, Miyagi 980-0845 Japan; 2 Hazaka Plant Research Center, Kennan Eisei Kogyo Co., Ltd., Sendai, Miyagi 989-1311 Japan; 3 Institute of Geophysics and Planetary Physics, Scripps Institution of Oceanography, University of California San Diego, La Jolla, CA 92093 USA; 4 Department of Chemistry, University of Washington, Box 351700, Seattle, WA 98195 USA

**Keywords:** Soil microbiology, Microbial ecology

Corrigendum to: *ISME Communications* 10.1038/s43705-022-00201-9, published online 16 December 2022

In the original version of the paper (page 12-13), the authors described the bacterial name *Vulcanimicrobium alpinus* (sic) gen. nov. sp. nov. along with higher-rank taxonomic names: the family (*Baltobacteraceae*), order (*Baltobacterales*), class (*Eremiobacteriia*), and phylum (*Eremiobacterota*). *Vulcanimicrobium alpinum* corrig. Yabe et al. 2023 was subsequently validated with a correction to the species name by Oren and Göker [1]. However, as the higher-rank taxonomic names and the nomenclatural type of a class contravene Rules 8, 9, and 15 of the *International Code of Nomenclature of Prokaryotes* [2], these names could not be validated. Therefore, the authors here provide the replacement names and protologues, *Vulcanimicrobiaceae* fam. nov., *Vulcanimicrobiales* ord. nov., *Vulcanimicrobiia* class. nov., and *Vulcanimicrobiota* phyl. nov. with following taxonomic descriptions.

Furthermore, in the Introduction of the original paper (page 1, right column, line 4), the authors inadvertently included *Actinomycetota*, which includes known photosynthetic lineages. However, the correct classification should have been *Acidobacteriota*.

The authors apologize for any confusion caused.

**Description of** ***Vulcanimicrobiaceae*** **fam. nov**.

(Vul.ca.ni.mi.cro.bi.a.ce’ae. N.L. neut. n. *Vulcanimicrobium*, a bacterial genus; -*aceae*, ending to denote a family; N.L. fem. pl. n. *Vulcanimicrobiaceae*, the *Vulcanimicrobium* family).

Not validly published synonym: "*Candidatus* Baltobacteraceae" [3, 4].

The description is the same as for the genus *Vulcanimicrobium*. Type genus is *Vulcanimicrobium* Yabe *et al*. 2023.

**Description of** ***Vulcanimicrobiales*** **ord. nov**.

(Vul.ca.ni.mi.cro.bi.a’les. N.L. neut. n. *Vulcanimicrobium*, a bacterial genus; -*ales*, ending to denote an order; N.L. fem. pl. n. *Vulcanimicrobiales*, the *Vulcanimicrobium* order).

Not validly published synonym: "*Candidatus* Baltobacterales" [3, 4].

The description is the same as for the genus *Vulcanimicrobium*. Type genus is *Vulcanimicrobium*.

**Description of** ***Vulcanimicrobiia*** **class. nov**.

(Vul.ca.ni.mi.cro.bi’i.a. N.L. neut. n. *Vulcanimicrobium*, a bacterial genus; -*ia*, ending to denote a class; N.L. pl. neut. n. *Vulcaniimicrobiia*, the *Vulcanimicrobium* class).

Not validly published synonyms: "*Candidatus* Eremiobacteriia", "*Candidatus* Eremiobacteria" [3, 5, 6].

The description is the same as for the genus *Vulcanimicrobium*. Type order is *Vulcanimicrobiales*.

**Description of** ***Vulcanimicrobiota*** **phyl. nov**.

(Vul.ca.ni.mi.cro.bi.o’ta. N.L. neut. n. *Vulcanimicrobium*, a bacterial genus; -*ota*, ending to denote a phylum; N.L. pl. neut. n. *Vulcaniimicrobiota*, the *Vulcanimicrobium* phylum).

Not validly published synonyms: "*Candidatus* Eremiobacteraeota", "*Candidatus* Eremiobacterota" [3, 5–7].

The description is the same as for the genus *Vulcanimicrobium*. Type genus is *Vulcanimicrobium*.
