# Supplementary material for: Vulcanimicrobium alpinus gen. nov. sp. nov., the first cultivated representative of the candidate phylum “Eremiobacterota”, is a metabolically versatile aerobic anoxygenic phototroph
Source: ISME Commun. 2022 Dec 16;2:120. doi: 10.1038/s43705-022-00201-9 (PMC9758169; doi:10.1038/s43705-022-00201-9)
Supplement: Supplementary file 1 — Supplementary Figures [file 43705_2022_201_MOESM1_ESM.pdf]

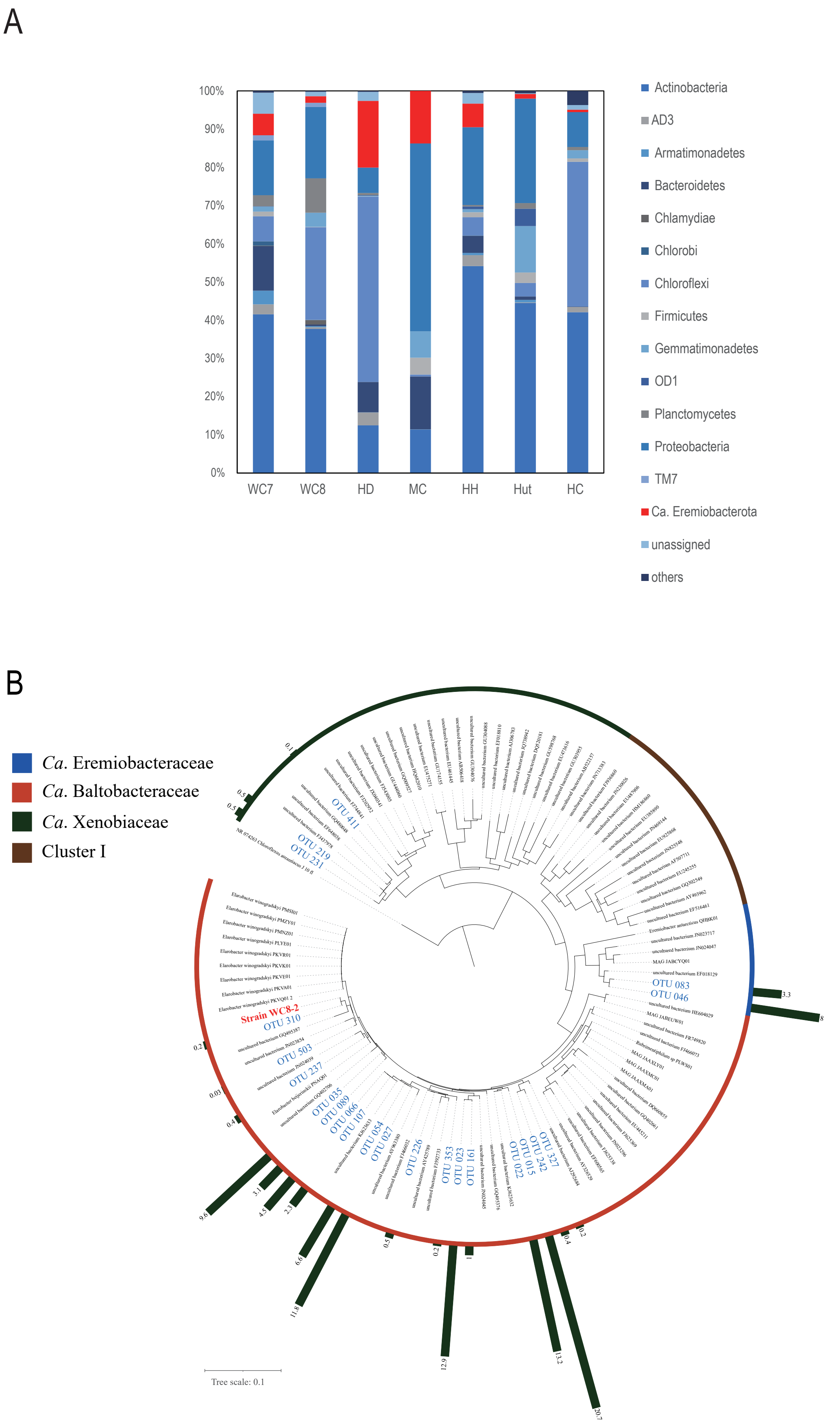

Supplementary Fig. S1. Distribution of *Ca*. Eremiobacterota community in the cave samples.
(a): Relative abundance of bacterial 16S rRNA gene amplicon reads derived from the seven cave samples. Taxa are displayed ≥1% read abundance in at least one sample. (b): Taxonomic distribution of *Ca*. Eremiobacterota OTUs in the samples, annotated with proportion of each OTU. The outer bar plots represent the relative abundance of each OTU.

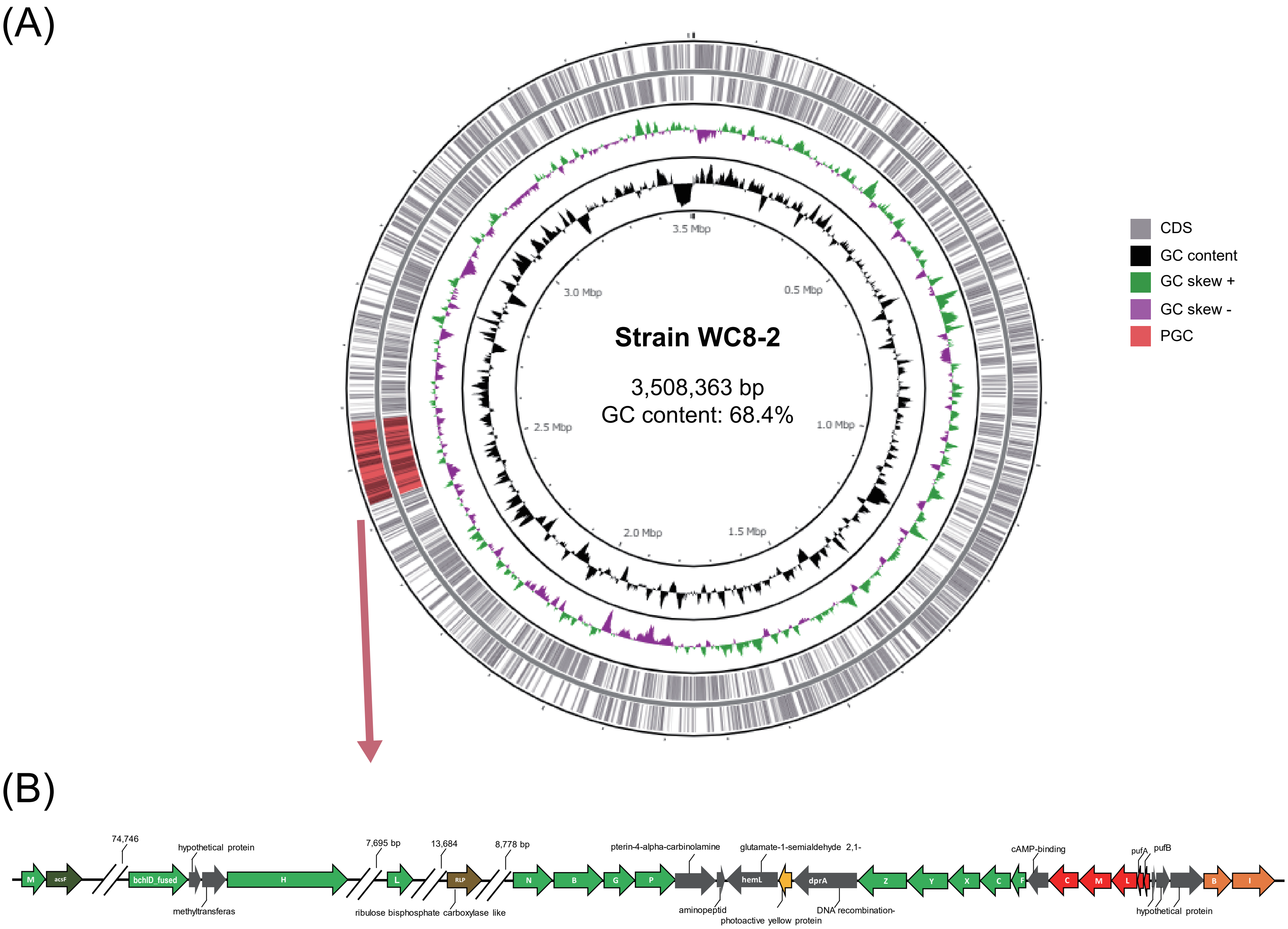

Supplementary Fig. S2. Genome and photosynthesis gene cluster of strain WPS-2.
(A) Circular genome of strain WC8-2. The black bars on the outer two circles show positions of protein-coding genes on plus and minus strands; the third circle is the value of GC-skew, and green and purple represent the leading and lagging strands, respectively. The fourth circle show GC content. The region highlighted in red represents the PGC. (B) The PGC in genome of strain WC8-2.

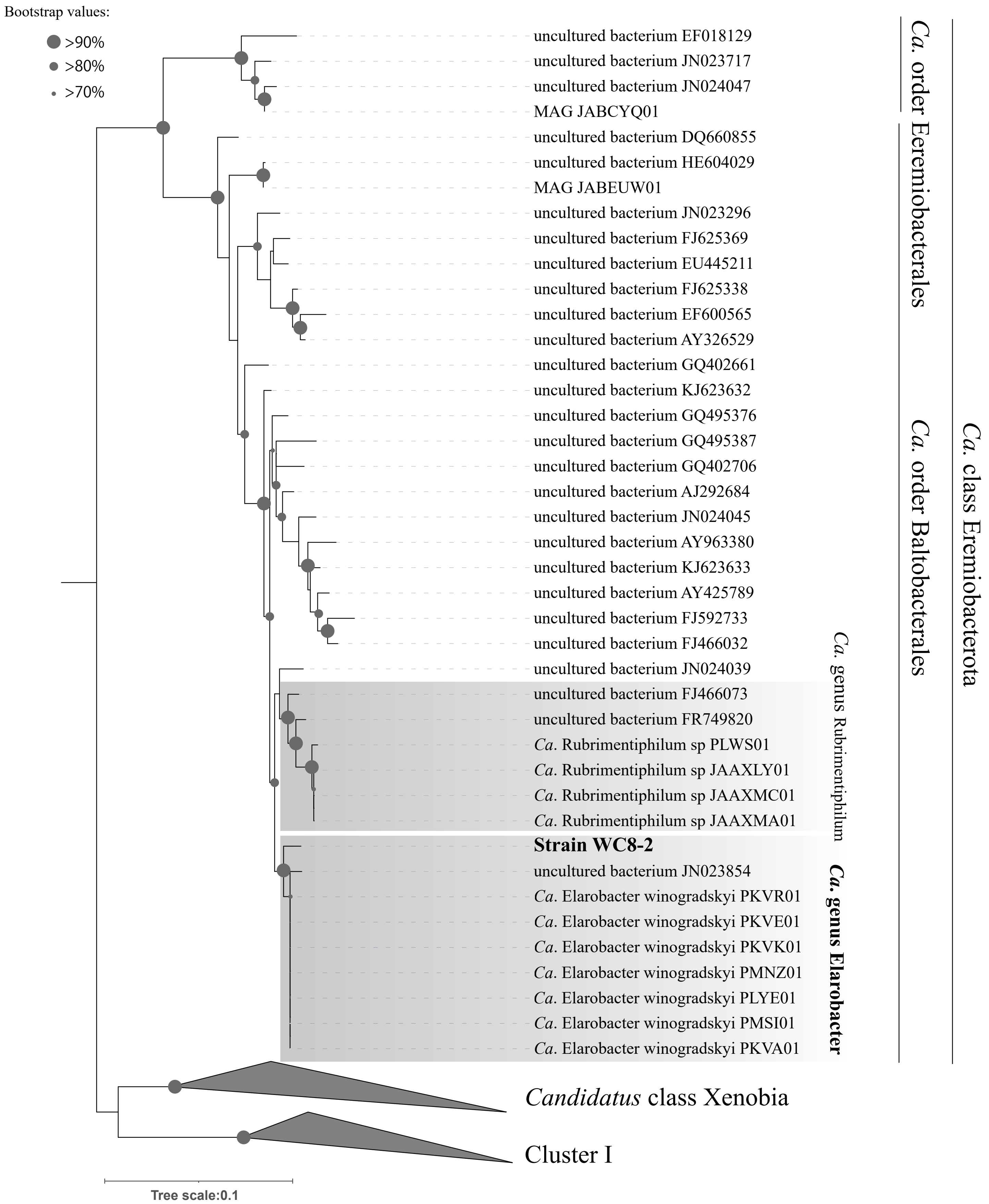

Supplementary Figure S3. Phylogenetic tree of strain WC8-2 within the phylum *Candidatus* Eremiobacterota based on near-full length 16S rRNA genes retrieved from the NCBI database and the MAGs. The phylogenetic tree was reconstructed using IQ-tree to produce maximum-likelihood trees with 1,000 bootstraps. Bootstrap support is shown as a solid gray circle for nodes with greater than 70% support with sizes proportional to the node supports. The scale bar represents substitutions per nucleotide base. *Chloroflexus aurantiacus* NR 074263 was set as the outgroup. The detailed information of the sequences in this tree are shown in Supplementary Table S2.

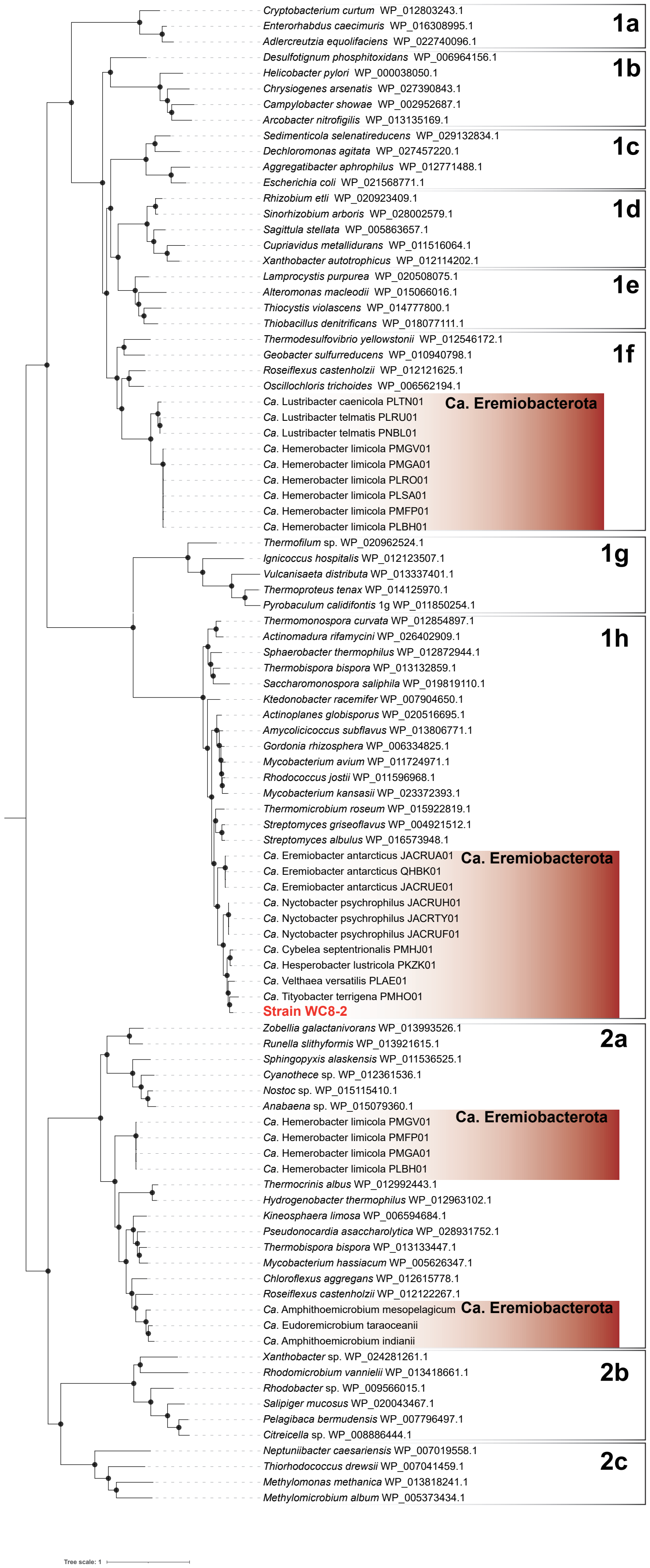

Supplementary Fig. S4. Maximum likelihood phylogeny of group I [NiFe]-hydrogenase large subunit (820 aligned positions).

The phylogenetic tree was reconstructed using IQ-tree with 1,000 bootstraps. Bootstrap support is shown as a solid black circle for nodes with greater than 80 % support. The phylogeny is midpoint rooted. The scale bar represents the number of amino acid substitutions per site.

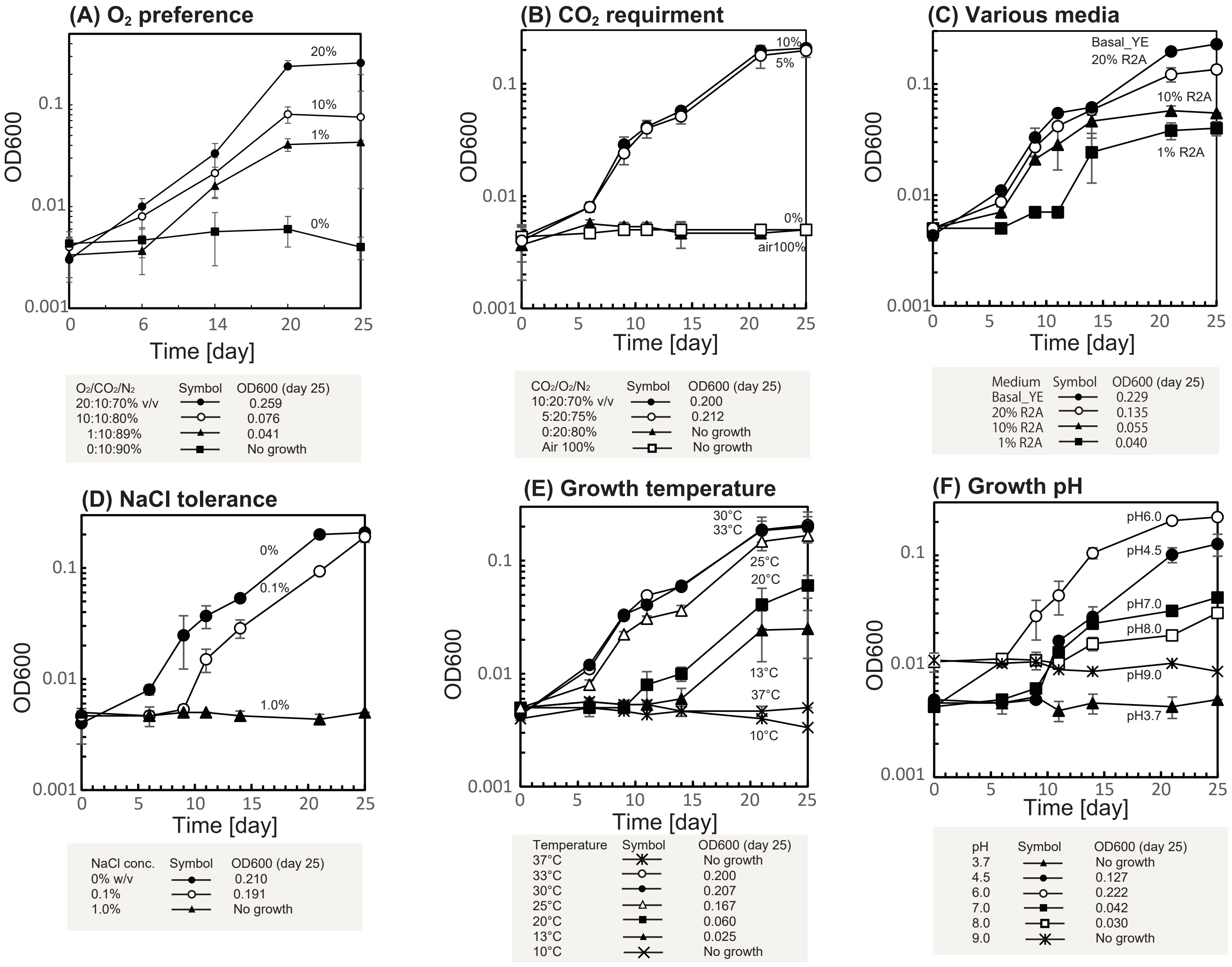

Supplementary Fig. S5. Growth of strain WC8-2 under various O_2 (A) and CO_2 (B) concentration, media (C), NaCl concentrations (D), Temperatures (E), and pH ranges (F)

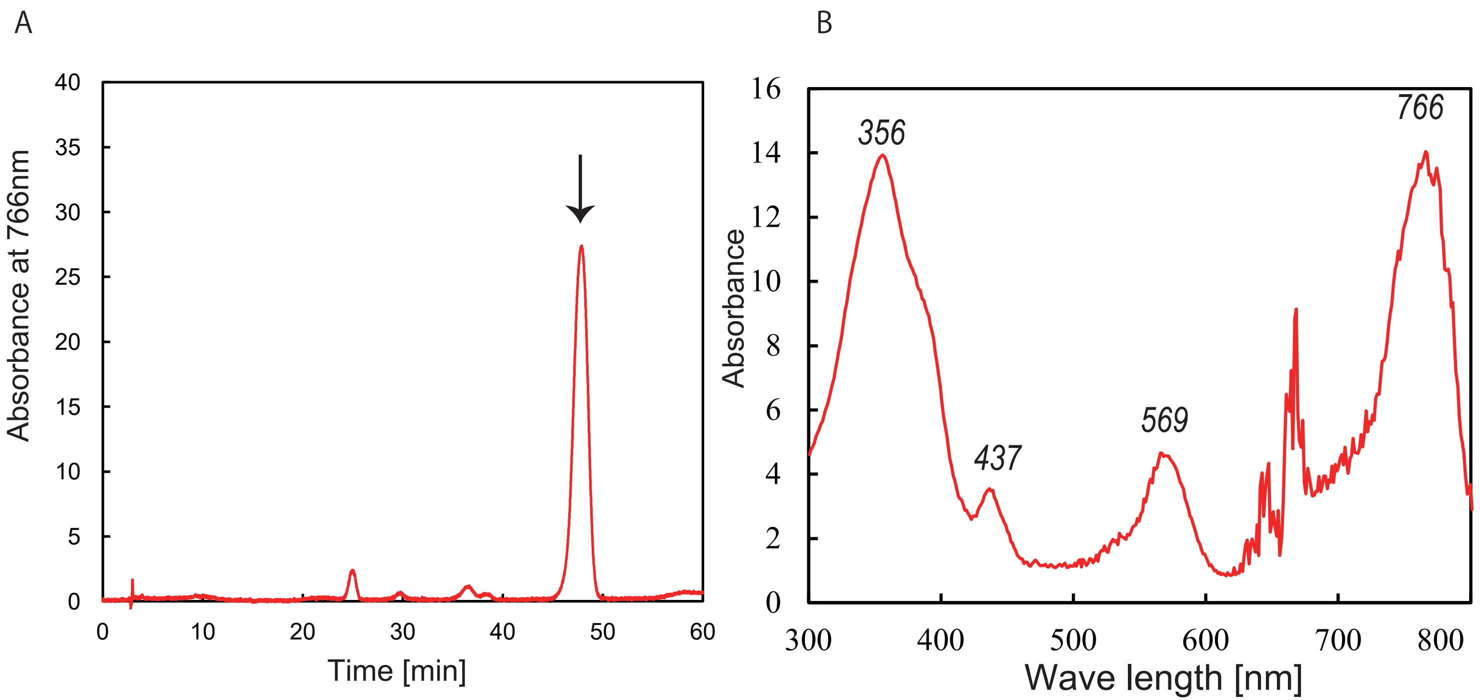

Fig.S6 Bacteriochlorophyll of strain WC8-2 separated by reversed-phase HPLC with monocratic elution using a 92.5% (v/v) methanol in water, monitered by a diode-array spectrophotometer detector.
(A) HPLC elusion profile monitored at 766 nm for BChl a. (B) The spectrum of the BChl *a* peak indicated by an arrow in (A).

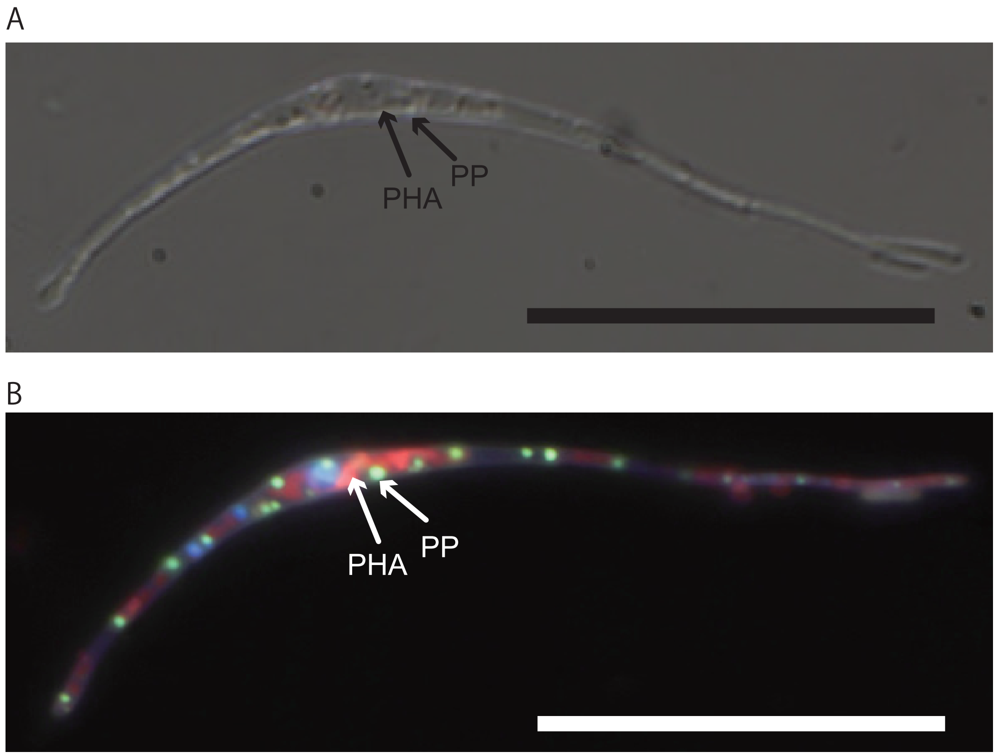

Supplementary Fig. S7. Differential interference contrast micrographs.
(A) Bright field; (B) Merged fluorescent images with the DAPI and Nile red (ex = 340-390 nm, em = 420 nm). Scale bars 20 μm.

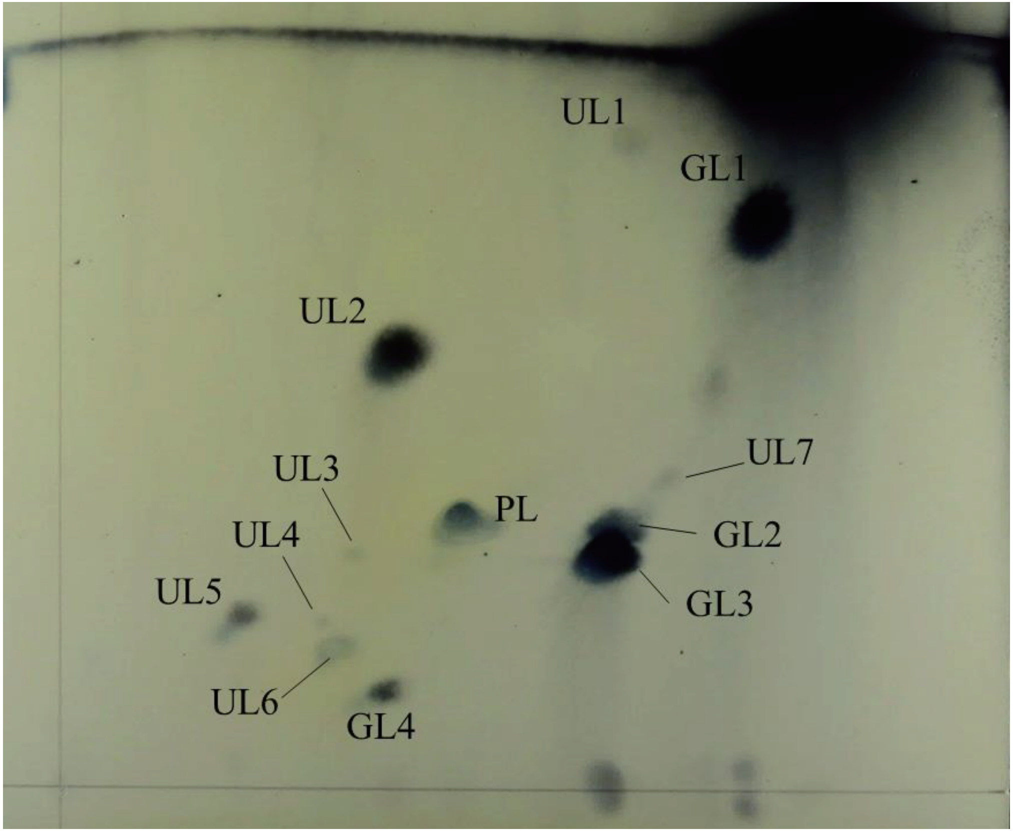

Fig. S8 The two-dimensional thin-layer chromatography of polar lipids visualized with phosphomolybdic acid. PL: unidentified phospholipid; GL: unidentified glycolipid; UL: unidentified lipid.
