# Supplementary material for: Vulcanimicrobium alpinus gen. nov. sp. nov., the first cultivated representative of the candidate phylum “Eremiobacterota”, is a metabolically versatile aerobic anoxygenic phototroph
Source: ISME Commun. 2022 Dec 16;2:120. doi: 10.1038/s43705-022-00201-9 (PMC9758169; doi:10.1038/s43705-022-00201-9)
Supplement: Supplementary file 2 — Supplementary Tables [file 43705_2022_201_MOESM2_ESM.pdf]

Supplementary Table S1 Cave Sample Information

Cave	Sample	Sample Type	Sample Date	Location	Light/dark	Sample temperature (°C)	Bacterial number (x 10^6/g)*
Warren Cave	WC1	Porous glassy material	12/03/10	S77.51639 E167.16708	Dark	No data	39.6
Warren Cave	WC2	Porous glassy material	11/26/12	S77.51639 E167.16708	Dark	No data	3.3
Warren Cave	WC3	Weathered basaltic/phonolitic sand	11/26/12	S77.51639 E167.16708	Dark	No data	12
Warren Cave	WC4	Weathered basaltic/phonolitic sand	11/26/12	S77.51639 E167.16708	Dark	No data	No data
Warren Cave	WC7	Pebbles and rock fragments	11/26/12	S77.51639 E167.16708	Dark	15	15.5
Warren Cave	WC8	Weathered basaltic/phonolitic sand	11/26/12	S77.51639 E167.16708	Dark	15	10.5
Warren Cave	WC10	Ash	11/26/12	S77.51639 E167.16708	Dark	No data	2.4
Harry's Dream	HD2	Weathered basaltic/phonolitic sand	11/24/12	S77.31.016 E 167.1308	Light	No data	No data
Harry's Dream	HD3	Weathered basaltic/phonolitic sand	11/24/12	S77.31.016 E 167.13087	Light	9	21.4
Mammoth Cave	MM	Pebbles and rock fragments	11/28/12	S77.52887 E167.19235	Dark	<5	7.2
Haggis Hole	HH	Pebbles and rock fragments	11/27/12	S 77.50961 E 167.13952	Light	7	1.9
Hut Cave	Hut	Weathered basaltic/phonolitic sand	12/2/12	S77.50893 E 167.14096	Light	9.2	3.1
Heroine Cave	HC	Porous glassy material	12/2/12	S 77.53261 E 167.11308	Light	10	1.8

*Bacterial numbers for the cave samples determined by quantitative PCR of small subunit ribosomal genes as described in ref [1]

References:

1. Tebo BM, Davis RE, Anitori RP, Connell LB, Schiffman P, Staudigel H. Microbial communities in dark oligotrophic volcanic ice cave ecosystems of Mt. Erebus, Antarctica. *Front Microbiol* 2015; **6**: 179.

Supplementary Table S4. Nucleotide sequences of PCR primers used to assay gene expressions by qRT-PCR.

Protein	Gene	Locus tag	Sequence, Tm (°C), GC content (%)		Amplicon size (bp)
			Forward primer	Reverse primer	
Reaction center protein L chain	*pufL*	WPS_26250	5’ -TTTCGTACTCCAGCCACCTC- 3’<br>Tm 60.25<br>GC 55.00	5’ -GTGAGGAAGAATCCGAGCAC- 3’<br>Tm 59.81<br>GC 55.00	106
Magnesium protoporphyrin IX methyltransferase	*bchM*	WPS_24950	5’ -CTGGACGTGCTGTTCCACTA- 3’<br>Tm 59.90<br>GC 55.00	5’ -GCATCCAGAAGTCGTTGAGC- 3’<br>Tm 60.96<br>GC 55.00	127
Ribulose bisphosphate carboxylase large chain	*cbbL*	WPS_15590	5’ -CGAGCTGCTCAACAAGTACG- 3’<br>Tm 59.81<br>GC 55.00	5’ -CCTTTGAGCGCTTCGTAGAC- 3’<br>Tm 60.15<br>GC 55.00	108
DNA-directed RNA polymerase subunit beta	*rpoB*	WPS_21400	5’ -GAAGAACGACGAGACCGAAG- 3’<br>Tm 59.99<br>GC 55.00	5’ -CGTCGTTTTCCCAGTTATGG- 3’<br>Tm 60.36<br>GC 50.00	122

Table S5. “Ca. Eremiobacterota” distribution in the fumarolic ice caves on Erebus volcano

(A) Relative abundance (%) of each phylum, (B) The eremiobacterial OTU composition in a "Ca. Eremiobacteria" community for each

(A)

	WC8	WC7	HD	MM	HH	Hut	HC
	Dark	Dark	Light	Dark	Light	Light	Light
Actinomycetota	**41.6%**	**37.7%**	**12.5%**	**11.4%**	**54.2%**	**44.5%**	**42.1%**
Ca. "Dormibacteriota"	2.6%	0.5%	3.4%	0.0%	2.9%	0.2%	1.4%
Armatimonadota	3.6%	0.3%	0.0%	0.0%	0.6%	0.7%	0.0%
Bacteroidota	**11.7%**	**0.4%**	**7.9%**	**13.8%**	**4.4%**	**0.9%**	**0.0%**
Chlamydiota	0.1%	1.2%	0.0%	0.0%	0.0%	0.0%	0.0%
Chlorobiota	1.2%	0.1%	0.0%	0.0%	0.1%	0.0%	0.0%
Chloroflexota	**6.5%**	**24.3%**	**48.5%**	**0.5%**	**4.8%**	**3.5%**	**37.9%**
Firmicutes	1.2%	0.1%	0.0%	4.4%	1.3%	2.8%	0.9%
Gemmatimonadota	**1.3%**	**3.7%**	**0.2%**	**6.9%**	**0.8%**	**12.2%**	**2.2%**
OD1	0.1%	0.0%	0.1%	0.0%	0.7%	4.5%	0.0%
Planctomycetota	2.9%	9.0%	0.6%	0.0%	0.4%	1.5%	0.8%
Proteobacteria	**14.3%**	**18.7%**	**6.6%**	**49.2%**	**20.4%**	**27.4%**	**9.1%**
TM7	1.3%	1.1%	0.0%	0.0%	0.0%	0.0%	0.0%
***Ca.* "Eremiobacterota"**	**5.6%**	**1.7%**	**17.4%**	**13.7%**	**6.2%**	**1.2%**	**0.6%**
unassigned	5.5%	1.1%	2.4%	0.0%	2.8%	0.2%	1.2%
others	0.5%	0.3%	0.3%	0.0%	0.6%	0.6%	3.7%

(B)

**#OTU ID**	***Candidatus* Family**	**WC8**	**WC7**	**HD3**	**MM**	**HH**	**Hut**	**HC**	**Total**
		**Dark**	**Dark**	**Light**	**Dark**	**Light**	**Light**	**Light**	
OTU_046	Eremiobacteraceae	59.3	4.3	0.0	0.0	0.0	0.0	0.0	8.0
OTU_083	Eremiobacteraceae	19.0	3.4	1.0	0.0	0.0	0.0	14.5	3.3
**total**		**78.3**	**7.6**	**1.0**	**0.0**	**0.0**	**0.0**	**14.5**	**11.3**
OTU_237	Baltobacteraceae	1.7	0.0	0.5	0.0	0.0	0.0	0.0	0.4
OTU_226	Baltobacteraceae	0.0	0.0	1.0	0.0	0.0	0.0	0.0	0.5
OTU_353	Baltobacteraceae	0.0	0.0	0.4	0.0	0.0	0.0	0.0	0.2
OTU_066	Baltobacteraceae	0.0	2.7	5.4	0.0	11.1	32.6	0.0	4.5
OTU_054	Baltobacteraceae	0.0	6.9	12.5	0.0	0.0	0.0	54.8	6.6
OTU_027	Baltobacteraceae	5.9	21.7	0.0	44.5	0.0	22.0	0.0	11.8
OTU_107	Baltobacteraceae	0.0	0.0	3.1	0.0	4.8	0.0	21.9	2.3
OTU_242	Baltobacteraceae	1.6	0.0	0.5	0.0	0.0	0.0	0.0	0.4
OTU_327	Baltobacteraceae	0.0	4.3	0.0	0.0	0.0	0.0	0.0	0.2
OTU_022	Baltobacteraceae	10.7	41.9	16.5	1.1	10.3	45.4	0.0	13.2
OTU_023	Baltobacteraceae	0.0	0.0	0.0	53.5	11.5	0.0	0.0	12.9
OTU_161	Baltobacteraceae	0.0	13.1	0.7	0.0	0.0	0.0	0.0	1.0
OTU_015	Baltobacteraceae	0.0	0.0	45.0	0.0	2.9	0.0	8.8	20.7
OTU_035	Baltobacteraceae	0.0	0.0	5.4	0.8	55.4	0.0	0.0	9.6
OTU_089	Baltobacteraceae	0.0	0.0	6.8	0.0	0.0	0.0	0.0	3.1
OTU_503	Baltobacteraceae	0.0	0.0	0.1	0.0	0.0	0.0	0.0	0.0
**OTU_310 (WC8-2)**	**Baltobacteraceae**	**1.8**	**0.0**	**0.0**	**0.0**	**0.0**	**0.0**	**0.0**	0.2
**total**		**21.7**	**90.6**	**97.9**	**100.0**	**96.0**	**100.0**	**85.5**	**87.6**
OTU_219	Xenobiaceae	0.0	0.0	0.0	0.0	4.0	0.0	0.0	0.5
OTU_231	Xenobiaceae	0.0	0.0	1.0	0.0	0.0	0.0	0.0	0.5
OTU_411	Xenobiaceae	0.0	1.8	0.0	0.0	0.0	0.0	0.0	0.1
**total**		**0.0**	**1.8**	**1.0**	**0.0**	**4.0**	**0.0**	**0.0**	**1.1**

Supplementary Table S6. Bacteria isolated from Warren cave and Harry's dream sediments, with phylogenetic placement to a bacterial phylum and identification of the closest cultivated neighbor.

Source	Colony name	Medium *	Phylum (class)	Closest cultivated species (Accession No.)	16S rRNA Identity; % (Total length; bp)	Accession No.
Warren cave						
WC1	WC1-1	R6.0_30	*Actinobacteria* (*Actinobacteria*)	*Microlunatus okinawensis* (LT629799)	98.01 (960)	LC668663
	WC1-3	R6.0_30	*Proteobacteria* (*a-proteobacteria*)	*Methylobacterium marchantiae* (FJ157976)	99.90 (1057)	LC668662
WC2	WC2-1	R6.0_30	*Actinobacteria* (*Actinobacteria*)	*Paenarthrobacter nicotinovorans* (X80743)	98.84 (447)	LC668669
	WC2-2	R4.5_30_az	*Actinobacteria* (*Actinobacteria*)	*Pseudarthrobacter chlorophenolicus* (CP001341)	98.85 (440)	LC668670
WC3	WC3-1	R6.0_30	*Proteobacteria*; (*g-proteobacteria*)	*Rhodanobacter umsongensis* (FJ821731)	98.88 (487)	LC668674
WC4	WC4-1	F6.0_30	*Firmicutes* (*Bacilli*)	*Tumebacillus lipolyticus* (KC462738)	91.56 (1399)	LC668697
	WC4-2	F6.0_30	*Firmicutes* (*Bacilli*)	*Tumebacillus lipolyticus* (KC462738)	90.24 (440)	LC668680
	WC4-3	F6.0_30	*Firmicutes* (*Bacilli*)	*Tumebacillus lipolyticus* (KC462738)	89.51 (456)	LC668681
	WC4-4	F6.0_30	*Firmicutes* (*Bacilli*)	*Tumebacillus lipolyticus* (KC462738)	89.83 (453)	LC668682
	WC4-5	R6.0_30	*Firmicutes* (*Bacilli*)	*Tumebacillus lipolyticus* (KC462738)	89.81 (434)	LC668683
	WC4-6	R6.0_30	*Firmicutes* (*Bacilli*)	*Tumebacillus lipolyticus* (KC462738)	90.39 (445)	LC668684
	WC4-7	R6.0_30	*Firmicutes* (*Bacilli*)	*Tumebacillus lipolyticus* (KC462738)	90.58 (447)	LC668685
	WC4-8	R6.0_30	*Firmicutes* (*Bacilli*)	*Tumebacillus lipolyticus* (KC462738)	89.86 (442)	LC668686
	WC4_9	R6.0_30	*Firmicutes* (*Bacilli*)	*Tumebacillus lipolyticus* (KC462738)	90.63 (433)	LC668687
	WC4_10	R6.0_30	*Firmicutes* (*Bacilli*)	*Tumebacillus lipolyticus* (KC462738)	90.42 (460)	LC668688
	WC4_11	R6.0_30	*Firmicutes* (*Bacilli*)	*Tumebacillus lipolyticus* (KC462738)	90.39 (449)	LC668689
	WC4_12	R6.0_30	*Firmicutes* (*Bacilli*)	*Tumebacillus lipolyticus* (KC462738)	90.42 (456)	LC668690
	WC4_13	R6.0_30	*Firmicutes* (*Bacilli*)	*Tumebacillus lipolyticus* (KC462738)	91.63 (1005	LC668691
	WC4_14	R6.0_30	*Firmicutes* (*Bacilli*)	*Tumebacillus lipolyticus* (KC462738)	91.51 (1048)	LC668692
	WC4_15	R6.0_30	*Firmicutes* (*Bacilli*)	*Tumebacillus lipolyticus* (KC462738)	91.53 (983)	LC668693
	WC4_16	R6.0_30	*Firmicutes* (*Bacilli*)	*Tumebacillus flagellates* (JQ421297)	97.02 (1051)	LC668678
	WC4_17	R6.0_30	*Firmicutes* (*Bacilli*)	*Tumebacillus lipolyticus* (KC462738)	90.61 (448)	LC668679
WC7	WC7-3	R6.0_15	*Actinobacteria* (*Actinobacteria*)	*Pseudarthrobacter enclensis* (JF421614)	98.28 (1048)	LC668654
	WC7-4	R6.0_15	*Actinobacteria* (*Actinobacteria*)	*Microlunatus okinawensis* (LT629799)	96.71 (1102)	LC668664
	WC7-5	R4.5_30	*Chloroflexi* (*Ktedonobacteria*)	*Thermosporothrix hazakensis* (AB500145)	84.97 (1119)	LC668661
	WC7-6	R4.5_30	*Proteobacteria* (*a -proteobacteria*)	*Sphingomonas aerophila* (KC735148)	99.13 (1040)	LC668675
	WC7_7	R6.0_30	*Proteobacteria*; (*b-proteobacteria*)	*Noviherbaspirillum psychrotolerans*（JN390675）	97.38 (888)	LC668666
	WC7_8	R6.0_30	*Proteobacteria*; (*b -proteobacteria*)	*Herbaspirillum autotrophicum*（LFLS01000071）	97.05 (1390)	LC668695
WC8	WC8-1	R6.0_15	*Actinobacteria* (*Actinobacteria*)	*Pseudarthrobacter enclensis* (JF421614)	98.20 (1061)	LC668655
	**WC8-2**	**R4.5_30**	***Candidatus* phylum Eremiobacterota**	***Thermosinus carboxydivorans* (AAWL01000046)**	**81.62 (1424)**	**LC579935**
WC10	WC10-1	R6.0_30	*Proteobacteria* (*a -proteobacteria*)	*Sphingomonas daechungensis* (JQ772481)	97.67 (1033)	LC668676
	WC10_2		*Firmicutes* (*Bacilli*)	*Streptococcus salivarius* (CP009913)	100.00 (1396)	LC668696
Harry's Dream						
HD2	HD2-1	R4.5_30_az	*Firmicutes* (*Bacilli*)	Anoxybacillus amylolyticus (AJ618979)	100.00 (476)	LC668653
HD3	HD3-1	R4.5_30_az	*Actinobacteria* (*Actinobacteria*)	*Pseudarthrobacter enclensis* (JF421614)	98.37 (1048)	LC668656
	HD3-2	R4.5_30_az	*Actinobacteria* (*Actinobacteria*)	*Pseudarthrobacter enclensis* (JF421614)	98.40 (1067)	LC668657
	HD3-3	R6.0_30	*Actinobacteria* (*Actinobacteria*)	*Pseudarthrobacter enclensis* (JF421614)	98.80 (999)	LC668658
	HD3-5	R6.0_30	*Actinobacteria* (*Actinobacteria*)	*Pseudarthrobacter chlorophenolicus* (CP001341)	98.87 (449)	LC668671
	HD3-6	R6.0_30	*Actinobacteria* (*Actinobacteria*)	*Pseudarthrobacter chlorophenolicus* (CP001341)	98.85 (451)	LC668672
	HD3-7	R6.0_30	*Actinobacteria* (*Actinobacteria*)	*Pseudarthrobacter chlorophenolicus* (CP001341)	98.85 (437)	LC668673
	HD3-13	R6.0_30	*Actinobacteria* (*Actinobacteria*)	*Paenarthrobacter nicotinovorans* (X80743)	98.62 (454)	LC668667
	HD3-14	R6.0_30	*Actinobacteria* (*Actinobacteria*)	*Paenarthrobacter nicotinovorans* (X80743)	98.58 (446)	LC668668
	HD3-16	R6.0_30	*Firmicutes* (*Bacilli*)	*Brevibacillus levickii* (AJ715378)	100 (1371)	LC668694
	HD3_20	R6.0_30	*Firmicutes* (*Bacilli*)	*Neobacillus fumarioli* (BCUZ01000224)	100.00 (473)	LC668665
	HD3_21	R6.0_30	*Firmicutes* (*Bacilli*)	*Neobacillus fumarioli* (BCUZ01000224)	100.00 (1369)	LC668699
	HD3_23	R6.0_30	*Firmicutes* (*Bacilli*)	*Bacillus cereus* (AE016877)	100.00 (489)	LC668659
	HD3_24	R6.0_30	*Firmicutes* (*Bacilli*)	*Bacillus circulans* (AY724690)	98.92 (476)	LC668660
	HD3_25	R6.0_30	*Firmicutes* (*Bacilli*)	*Streptococcus salivarius* (CP009913)	100.00 (474)	LC668677
	HD3_27	F6.0_37	*Deinococcus-Thermus* (*Deinococci*)	*Meiothermus cerbereus* (JHVI01000018)	92.00 (1438)	LC668698

* Medium abbreviations: The first capital letter R or F indicates the type of medium, R for 10% R2A gellan gum medium, F for FS1V gellan gum medium, the following numbers 4.5 or 6.0 indicate the pH of the medium, and the following numbers 15 or 30 indicate the incubation temperature. If "az" is mentioned at the end of a word, it indicates that the medium contains 30 mg/L of sodium azide.

Table S7. Amino acid identities (AAI) between strain WC8-2 and representative species in each class within the phylum *Armatimonadota*, *Chloroflexota*, and Abditibacterota.

Values were calculated based on the total number of proteins using the AAI calculator at (http://enve-omics.ce.gatech.edu/).

No.	Phylum	Representative in each class	1	2	3	4	5	6	7	8	9	10	11	12
1	*Eremiobacterota*	Strain WC8-2	**100.0**											
2	*Armatimonadota*	*Armatimonas rosea* YO-36	**38.8**	100.0										
3		*Fimbriimonas ginsengisoli* Gsoil348	**38.6**	42.2	100.0									
4	*Chloroflexota*	*Anaerolinea thermolimosa* IMO1	**38.5**	38.6	38.0	100.0								
5		*Ardenticatena maritima* 110S	**40.4**	39.7	38.8	45.5	100.0							
6		*Caldilinea aerophila* DSM14535	**39.7**	39.2	38.4	46.0	48.3	100.0						
7		*Chloroflexus aggregans* DSM9485	**40.4**	39.4	38.5	43.1	46.6	46.5	100.0					
8		*Dehalococcoides mccartyi* 195	**38.3**	38.4	37.7	40.5	41.4	40.0	40.8	100.0				
9		*Thermoflexus hugenholtzii* JAD2	**40.7**	39.4	38.8	47.2	49.3	47.1	45.4	41.4	100.0			
10		*Thermomicrobium roseum* DSM5159	**41.2**	39.5	39.1	42.2	45.4	43.0	44.7	41.2	46.1	100.0		
11		*Thermosporothrix hazakensis* SK20-1	**39.4**	38.3	37.8	40.3	42.5	41.2	41.8	40.3	42.1	42.3	100.0	
12	*Abditibacterota*	*Abditibacterium utsteinense* LMG29911	**38.7**	40.5	39.7	38.1	38.8	38.5	38.9	38.2	38.9	38.8	38.0	100.0

Table S9. Growth of strain WC8-2 under (A) photo- and chemo- organoheterotrophic and (B) photo- and chemo- lithoautotrophic conditions, and (C) anaerobic/fermentative conditions.

Unless otherwise noted, all cultures were grown in 100 mL butyl stopper- and screw-cap-sealed glass vials containing 50 ml Basal_YE (pH6.0) at 30°C with air/CO_2 (90:10, v/v).

(A) Photo- and chemo- organoheterotrophic growth

	Continuous light		Continuous dark	
Basal_medium supplemented with	Aerobic	Anaerobic	Aerobic	Anaerobic
0.03% (v/v) glycerol	-	-	-	-
0.03% (w/v) sucrose	-	-	-	-
0.03% (w/v) D-glucose	-	-	-	-
0.03% (w/v) D-ribose	-	-	-	-
0.03% (w/v) maltose	-	-	-	-
0.03% (w/v) L-leucine	-	-	-	-
0.03% (w/v) L-isoleucine	-	-	-	-
0.03% (w/v) L-valine	-	-	-	-
0.03% (w/v) L-serine	-	-	-	-
0.03% (w/v) L-lysine	-	-	-	-
0.03% (w/v) taurine	-	-	-	-
0.03% (w/v) yeast extract (See Fig. 4)	+	-	+	-
0.03% (w/v) gellan gum	-	-	-	-
0.1% (v/v) vitamin B12 solution (2mg/L)	-	-	-	-

(B) Photo- and chemo- lithoautotrophic growth

	Continuous light		Continuous dark	
Amended PSB2 and Basal_medium supplemented with (as an electron donor)	Aerobic	Anaerobic	Aerobic	Anaerobic
5 mM Na_2S	-	-	-	-
5 mM $Na_2S_2O_3$	-	-	-	-
1% H_2 (v/v; in the gas phase)	-	-	-	-

(C) Anaerobic/Fermentative growth

20% R2A supplemented with (as an electron acceptor)	Continuous dark/Anaerobic condition
5 mM Na_2SO_4	-
5 mM $NaNO_3$	-
5 mM DMSO	-

Table S10. Comparison of puf, bchM, and cbbL gene expression levels using qPCR performed with different dilutions of cDNA in the light

Gene	Light/Dark		cal_curve 1	cal_curve 2	cal_curve 3	Mean	SD	t-test
Reference control rpoB	Light	intercept (Ct)	23.2	23.1	23.4			
		efficiency	0.97	0.97	0.98	97%	0.2%	
	Dark	intercept (Ct)	23.4	23.6	23.9			
		efficiency	0.98	0.99	0.98	98%	0.4%	
pufL	Light	intercept (Ct)	23.3	23.3	23.6			
		efficiency	0.97	0.97	0.97	97%	0.1%	
		Δct (pufL-rpoB)	0.18	0.12	0.27			
	Dark	intercept (Ct)	24.4	24.5	25.3			
		efficiency	0.95	0.95	0.96	95%	0.4%	
		Δct (pufL-rpoB)	0.98	0.88	1.38			
		ΔΔct (Δct_light-Δct_dark)	-0.80	-0.76	-1.11			
		**2-ΔΔct**	**1.74**	**1.69**	**2.16**	**1.86**	**0.21**	**0.00512**
bchM	Light	intercept (Ct)	25.5	26.0	25.8			
		efficiency	0.95	0.95	0.96	95%	0.2%	
		Δct (bchM-rpoB)	2.30	2.82	2.41			
	Dark	intercept (Ct)	27.6	27.8	27.6			
		efficiency	0.97	0.98	0.98	97%	0.7%	
		Δct (bchM-rpoB)	4.16	4.21	3.75			
		ΔΔct (Δct_light-Δct_dark)	-1.75	-1.39	-1.45			
		**2-ΔΔct**	**3.37**	**2.62**	**2.73**	**2.90**	**0.33**	**0.00203**
cbbL	Light	intercept (Ct)	20.3	20.7	21.2			
		efficiency	0.99	0.99	0.99	99%	0.0%	
		Δct (cbbL-rpoB)	-2.90	-2.42	-2.16			
	Dark	intercept (Ct)	20.3	21.4	21.4			
		efficiency	0.98	0.98	0.98	98%	0.2%	
		Δct (cbbL-rpoB)	-3.05	-2.17	-2.49			
		ΔΔct (Δct_light-Δct_dark)	0.15	-0.25	0.33			
		**2-ΔΔct**	**0.90**	**0.99**	**0.95**	**0.95**	**0.04**	**0.82777**

Table S11 Cellular fatty acids composition (%) of strain WC8-2

$C_{9:0}$	0.28
$C_{10:0}$	0.23
$C_{12:0}$ 3OH	1.05
$C_{13:0}$ 2OH	0.7
$C_{14:0}$	0.92
$C_{16:0}$	1.17
$C_{18:0}$	1.28
$C_{19:0}$	0.13
$C_{20:0}$	0.32
iso-$C_{11:0}$	1.04
iso-$C_{12:0}$	0.81
iso-$C_{12:0}$ 3OH	1.23
iso-$C_{13:0}$	0.2
iso-$C_{13:0}$ 3OH	10.95
iso-$C_{14:0}$	33.92
iso-$C_{14:0}$ 3OH	16.49
iso-$C_{15:0}$	1.47
anteiso-$C_{15:0}$	2.07
iso-$C_{16:0}$	7.41
iso-$C_{17:0}$	1.64
anteiso-$C_{17:0}$	0.75
$C_{17:0}$ cyclo	0.33
iso-$C_{17:0}$ 3OH	2.25
iso-$C_{18:0}$	0.7
$C_{17:0}$ 10-methyl	0.72
$C_{14:1}\omega 5c$	3.92
iso-$C_{15:1}$ F	0.13
$C_{16:1}$ $\omega 5c$	0.25
$C_{16:1}$ 2OH	0.92
iso-$C_{18:1}$ H	0.19
iso-$C_{18:1}$ $\omega 9c$	1.09
Summed feature 1*	0.46
Summed feature 2*	0.65
Summed feature 6*	0.36
Summed feature 7*	3.96

*Summed feature 1, iso-C15:1 H and/or C13:0 3-OH; summed feature 2, one or more of C12:0 aldehyde, unknown ECL 10.928, iso-C16:1 I and C14:0 3-OH; summed feature 6, C19:1*ω11c* and/or C19:1*ω9c*; summed feature 7, one or more of unknown ECL18.846, $C_{19:1}\omega 6c$, $C_{19:0}$ cyclo $\omega 10c$ and $C_{19:1}\omega 6c$.
